# Premetazoan Origin of Neuropeptide Signaling

**DOI:** 10.1093/molbev/msac051

**Published:** 2022-03-12

**Authors:** Luis Alfonso Yañez-Guerra, Daniel Thiel, Gáspár Jékely

**Affiliations:** Living Systems Institute, University of Exeter, Stocker Road, Exeter, United Kingdom

**Keywords:** neuropeptide, nesfatin, phoenixin, choanoflagellate, sponge, ctenophore

## Abstract

Neuropeptides are a diverse class of signaling molecules in metazoans. They occur in all animals with a nervous system and also in neuron-less placozoans. However, their origin has remained unclear because no neuropeptide shows deep homology across lineages, and none have been found in sponges. Here, we identify two neuropeptide precursors, phoenixin (PNX) and nesfatin, with broad evolutionary conservation. By database searches, sequence alignments, and gene-structure comparisons, we show that both precursors are present in bilaterians, cnidarians, ctenophores, and sponges. We also found PNX and a secreted nesfatin precursor homolog in the choanoflagellate *Salpingoeca rosetta*. PNX, in particular, is highly conserved, including its cleavage sites, suggesting that prohormone processing occurs also in choanoflagellates. In addition, based on phyletic patterns and negative pharmacological assays, we question the originally proposed GPR-173 (SREB3) as a PNX receptor. Our findings revealed that secreted neuropeptide homologs derived from longer precursors have premetazoan origins and thus evolved before neurons.

## Introduction

Neuropeptides are one of the largest families of neuronal signaling molecules. They are derived from precursor molecules (proneuropeptides) that must undergo processing to release active mature peptides (Veenstra 2000). Neuropeptides play pivotal roles in the regulation of different biological processes such as feeding, cognition, and reproduction, and they have been extensively studied in bilaterians (Jékely 2013; Mirabeau and Joly 2013; Elphick et al. 2018; Thiel et al. 2021). Neuropeptide-like molecules have also been described in cnidarians, ctenophores, and the neuron-less placozoan *Trichoplax adhaerens* (Takahashi et al. 2008; Jékely 2013; Senatore et al. 2017; Varoqueaux et al. 2018; Koch and Grimmelikhuijzen 2020; Takahashi 2020; Burkhardt and Jékely 2021; Sachkova et al. 2021). So far, none of the neuropeptides described in these species has shown enough similarity to be considered direct orthologs of bilaterian short neuropeptides. However, some short peptides in cnidarians have been suggested to be potential one-to-many orthologs of bilaterian neuropeptides. These include cnidarian GLWamides potentially related to bilaterian Wamide neuropeptides (Jékely 2013; Williams 2020) and the hydrozoan maturation-inducing hormones (MIHs) with an identified receptor that is a one-to-many homolog of bilaterian neuropeptide Y, neuropeptide FF, tachykinin, orexin, elevenin, EFLGa/thyrotropin-releasing hormone, and luqin receptors (Quiroga Artigas et al. 2020). Potential ctenophore homologs of bilaterian large cysteine-rich hormones including trunk-like proteins and prothoracicotropic hormone have also been identified (de Oliveira et al. 2019). In addition, sponges have cystine-knot family growth factors, related to bilaterian glycoprotein hormones (Roch and Sherwood 2014).

Despite the lack of deep conservation in neuropeptides, the machinery involved in precursor processing had evolved before metazoans. Homologs of peptidyl-glycine α-amidating monooxygenase, an enzyme important for the amidation of neuropeptides in Bilateria, and prohormone convertases, involved in the proteolytic cleavage of proneuropeptides exist in the green algae *Chlamydomonas reinhardtii* and other ciliated protists (Kumar et al. 2016, 2017; Luxmi et al. 2018, 2019). In *C. reindhardtii*, several amidated peptides have been identified, and some have signaling functions during gamete chemotaxis (Luxmi et al. 2019). These results suggest that neuropeptide signaling in metazoans has a deep evolutionary ancestry in single-celled eukaryotes. However, the *C. reindhardtii* peptides show no similarity to any metazoan neuropeptides, and it remains unclear when animal neuropeptides evolved.

Here, we report two neuropeptide precursor sequences of premetazoan origin—the phoenixin (PNX) and nesfatin precursors—with orthologs in all major metazoan branches, as well as choanoflagellates. PNX was first identified by screening the human genome database (Yosten et al. 2013a). The mature PNX peptide is derived from the PNX precursor, also named small integral membrane protein 20 (SMIM20). The PNX precursor is highly conserved across vertebrates and contains a signal peptide and dibasic cleavage sites (Yosten et al. 2013a). In mammals, this precursor undergoes post-translational processing to produce two alternative C-terminally amidated peptides, PNX-14 and PNX-20, with PNX-14 being the most abundant peptide in rodent tissues (Lyu et al. 2013; Yosten et al. 2013a). This peptide is mainly expressed in the central nervous system, in the hypothalamus, the central nucleus of the amygdala, and the supraoptic nucleus. Expression was also detected in peripheral tissues including the pancreas and the small intestine (Prinz et al. 2017). Some vertebrate PNX precursors lack the amidation signature in the C-terminal PNX region, including the *Xenopus*, *Silurana*, zebrafish, and fugu precursors (Yosten et al. 2013a). Recent searches also identified arthropod PNX precursors with conserved cleavage sites to produce the predicted mature PNX peptides, PNX-14 and PNX-20 (Nguyen et al. 2018).

Pharmacological experiments with the mature PNX peptides suggest that PNX-14 and PNX-20 act as pleiotropic neuropeptides in mammals. PNX peptides modulate heart function, memory, anxiety, food intake, and reproduction (Clarke and Dhillo 2019; Schalla and Stengel 2019; Billert et al. 2020; Haddock et al. 2020; Ma et al. 2020; Schalla et al. 2020; Friedrich and Stengel 2021; Yao et al. 2021). Strikingly for a proneuropeptide, the SMIM20 (also called MITRAC7) precursor also localizes to mitochondria in U2OS and HEK293 cells and functions as a mitochondrial chaperone during cytochrome-c oxidase complex assembly (Dennerlein et al. 2015). These observations suggest that the PNX/SMIM20 precursor is a moonlighting protein, being targeted to different cellular localizations and exhibiting different biochemical functions within the same polypeptide chain (Jeffery 2018).

The mechanism of signaling by the processed PNX neuropeptides remains unclear. However, the receptor GPR173, also known as Super conserved Receptor Expressed on Brain 3 (SREB3), has been proposed as a potential receptor of PNX. This proposal was based on a “Deductive Reasoning Strategy,” a patented methodology that has been described for some neuropeptide–receptor pairings (e.g., neuronostatin–GPR107) but not thoroughly explained in the case of the proposed PNX–GPR173 pairing (Stein et al. 2016; Yosten et al. 2021). Although this strategy was successful in predicting one ligand–receptor pair (neuronostatin–GPR107), another prediction could not be confirmed experimentally (proinsulin C–GPR146) (Yosten et al. 2013b; Lindfors et al. 2020).

Nucleobindin-2-Encoded Satiety and FAT-Influencing proteiN-1 (Nesfatin-1) is a neuropeptide identified in 2006 as an 82 amino acid peptide located in the N-terminal region of the protein nucleobindin-2 (NUCB2) (Oh-I et al. 2006). NUCB precursors contain a signal peptide, dibasic cleavage sites, and leucine zipper and EF-hand motifs. It has been shown that these precursors can act as calcium and DNA-binding proteins in addition to producing mature neuropeptides (Miura et al. 1992, 1994; Kmiecik et al. 2021). NUCB precursors encode three different potential peptides, known as nesfatin-1, 2, and 3. So far, only nesfatin-1 was shown to have a physiological function (Oh-I et al. 2006; Schalla and Stengel 2018). Different functions of nesfatin-1 have been reported in vertebrates, including the regulation of glucose metabolism, reproduction, anxiety, and responses to stress (Schalla and Stengel 2018; Friedrich and Stengel 2021). Its role as a satiety-inducing factor has been widely reported in mammals and fish (Ayada et al. 2015; Sundarrajan et al. 2016; Rupp et al. 2021). NUCB precursors encoding the nesfatin-1 peptide region are also present in invertebrates, including ophiuroid echinoderms and the fruit fly *Drosophila melanogaster* (Otte et al. 1999; Zandawala et al. 2017). So far, no receptor has been identified for the nesfatin-1 peptide (Rupp et al. 2021).

Here, we present a comprehensive bioinformatic survey of the neuropeptide precursors, PNX and nesfatin, in bilaterian and nonbilaterian animals, as well as different unicellular eukaryotes. Using sequence-similarity searches, alignments, and gene-structure analyses, we identified an ancient origin of these neuropeptide precursors. Our findings indicate that some of the secreted neuropeptide-like molecules have deep origins and evolved before nervous systems. These findings support a model for the stepwise assembly of neuronal signaling systems from pre-existing components at the origin of nervous systems.

## Results and Discussion

### PNX and Nesfatin-1 Precursors Were Already Present in the Last Common Ancestor of Metazoa and Choanoflagellates

In an initial bioinformatic survey, we noticed that nesfatin and PNX have a broad phyletic distribution in animals and their closest protistan relatives, suggesting that these may be the oldest neuropeptides found so far. To analyze this in more detail, we searched the transcriptome and genome sequences of 45 metazoan species (supplementary file S1 and fig. S1, Supplementary Material online), two choanoflagellates, one filasterean, and *Tunicaraptor unikontum*, a predatory flagellate belonging to a newly identified animal-related lineage (Tikhonenkov et al. 2020). By sequence-similarity searches, multiple-sequence alignments, and gene-structure analyses, we identified homologs of the precursors of PNX (SMIM20) and nesfatin-1 (NUCB) across major groups of metazoans including Porifera, Ctenophora, Cnidaria, and in most of the bilaterian species analyzed. We could not identify either peptide in placozoans. The PNX precursor seems to be absent in placozoans, and in the case of the nesfatin-1, we could identify a NUCB precursor. However, the region containing the nesfatin-1 peptide is missing in this placozoan NUCB homolog. Finally, a PNX precursor was identified in the choanoflagellate *Salpingoeca rosetta*, and a NUCB homolog was detected in *S. rosetta* and *T. unikontum*, with both missing the nesfatin-1 peptide. We could not find related sequences in any other eukaryotes more distantly related to animals. Overall, these findings reveal that the PNX and nesfatin neuropeptide precursors originated in premetazoan times.

### The PNX Precursor is Highly Conserved in Metazoans and Choanoflagellates

**Fig. 1.**
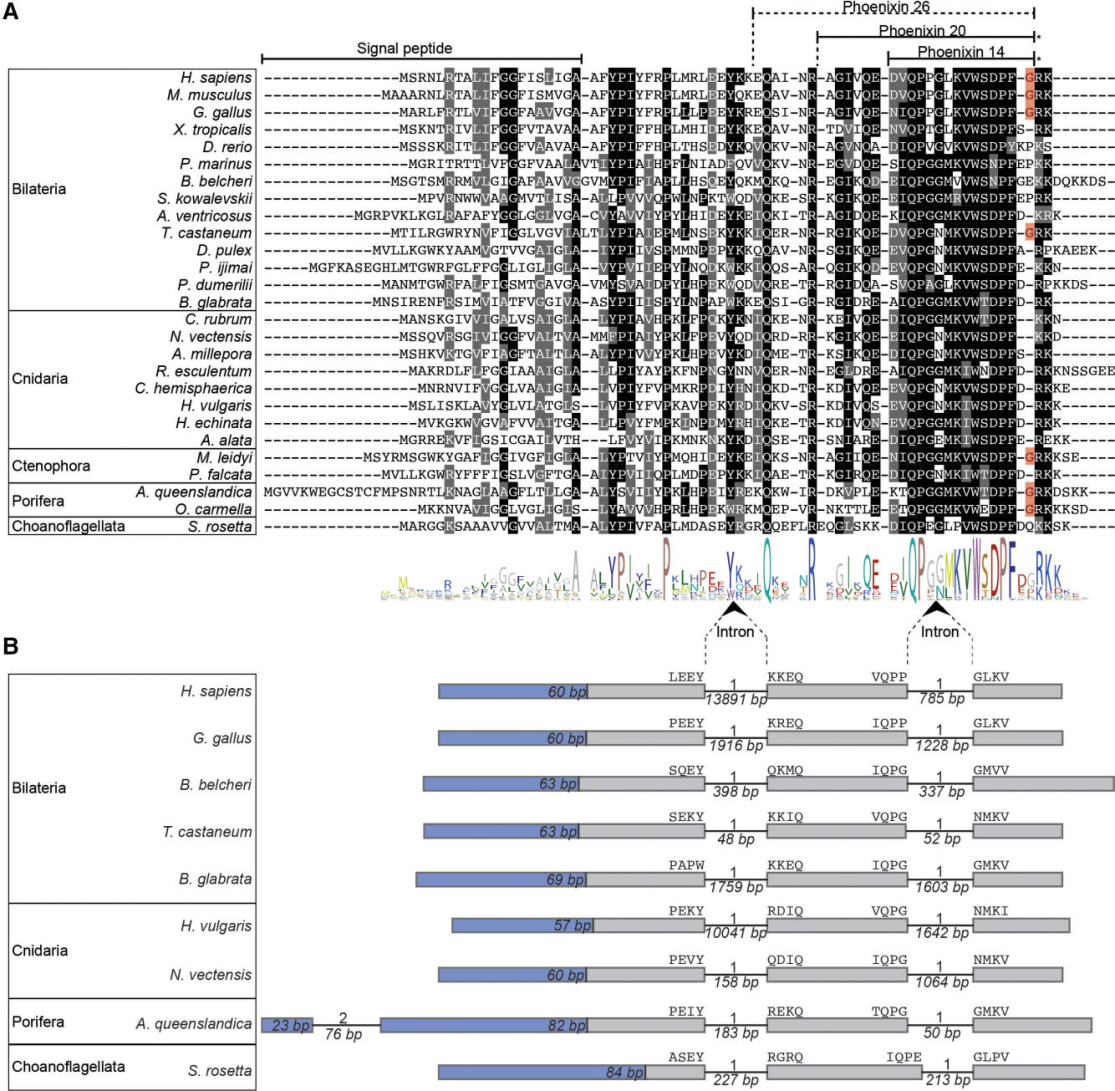
Sequence alignment and genomic structure of PNX precursors. (*A*) Alignment of the PNX precursors containing the PNX peptides. Predicted signal peptides and mature peptides are indicated with lines. Residues that are conserved in more than 50% of the sequences are shown in black, and conservative substitutions are shown in gray. Amidation sites are highlighted in red. (*B*) Exon–intron structure of PNX precursor genes. The regions encoding the signal peptides are in blue with their length indicated. Amino acids encoded at the exon–intron junctions are shown above the exon boxes. Introns are shown as lines, and their length in base pairs is indicated below. The intron phase is shown above the introns.

A multiple-sequence alignment reveals a high degree of conservation of the PNX neuropeptide precursors (fig. 1*A*). There are widely conserved residues across the length of the sequences, with the C-terminal region—corresponding to the mammalian PNX-14 peptide (Lyu et al. 2013; Yosten et al. 2013a)—being the most conserved across all tested species (with IQPGGMKVWSDPFD as the consensus sequence). The regions that contain the dibasic and monobasic cleavage sites for proteolytic processing (Veenstra 2000; Hook et al. 2008) are also well conserved across metazoans and in *S. rosetta* (fig. 1*A*).

The predicted neuropeptides show variation in the C-terminal amidation site across metazoans, similar to the variability in C-terminal amidation that was already described within vertebrates (Yosten et al. 2013a). Within the deuterostomes, only some vertebrates possess an amidation site. In the protostome sequences analyzed, only the *Tribolium castaneum* PNX has an amidation signature, whereas other protostome PNXs do not. This suggests that the ancestral bilaterian PNX was not amidated, and the amidation site found in some vertebrates evolved convergently to the one present in *T. castaneum*. None of the cnidarian PNXs show an amidation site. Within Ctenophora, the *Mnemiopsis leidyi* peptide is predicted to be amidated, whereas the *Pukia falcata* PNX lacks the amidation motif. The *Oscarella carmella* and *Amphimedon queenslandica* sponge PNX precursors have an amidation site but the choanoflagellate *S. rosetta* precursor lacks it. Based on this phyletic pattern, it is not possible to say whether the ancestral metazoan PNX was amidated or not. In either scenario, however, there have been several convergent gains or losses of the PNX amidation site during metazoan evolution. Overall, an amidation site is absent from the majority of the sequences studied here, and many show an acidic residue (D, E) instead (fig. 1*A*). In most other neuropeptide families, the C-terminal amidation of homologous peptides is conserved across species (Mirabeau and Joly 2013; Sobrido-Cameán et al. 2020; Yañez-Guerra et al. 2020). However, there are some examples where this is not the case, such as galanin, which is amidated in most vertebrates but not in humans (Sobrido-Cameán et al. 2019). This non-amidated version of human galanin is, nevertheless, functional (Bersani et al. 1991). Thus, it is possible that the amidation of PNX, just like in the case of galanin, is important only in certain species.

To further test the homology of PNX neuropeptides, we carried out a gene-structure analysis of the *Homo sapiens* (Mammalia), *Gallus gallus* (Sauropsida), *Branchiostoma belcheri* (Cephalochordata), *T. castaneum* (Ecdysozoa), *Biomphalaria glabrata* (Lophotrochozoa), *Nematostella vectensis* (Cnidaria), *Hydra vulgaris* (Cnidaria), *A. queenslandica* (Porifera), and *Salpingoeca rosetta* (Choanoflagellata) PNX precursor genes. This revealed a high level of conservation of these precursors at the gene-structure level within and beyond metazoans. Most of these proteins are encoded in three exons, divided by two phase-1 introns at homologous positions (fig. 1*B*). In the sponge *A. queenslandica*, there is an additional intron in the region encoding the signal peptide (fig. 1*B*). The conservation of sequence and gene structure unambiguously demonstrates the homology of the PNX precursors.

The signal peptide of the PNX precursors is unusual. We could only detect it by SignalP3.0 (Bendtsen et al. 2004) and not by SignalP4.1 (Petersen et al. 2011), SignalP5.0 (Almagro-Armenteros et al. 2019b), or SignalP6.0 (Teufel et al. 2022). These newer versions of the software failed to identify the signal peptide even in the human PNX precursor protein where propeptide cleavage has been experimentally demonstrated (Yosten et al. 2013a) or in the PNX precursor from *N. norvegicus* (Nguyen et al. 2018). To further explore this, we used the machine-learning–based tools DeepLoc1.0 (Almagro-Armenteros et al. 2017) and TargetP2.0 (Almagro-Armenteros et al. 2019a) developed to predict the subcellular localization of protein sequences. Again, we found conflicting results, suggesting either that the PNX precursors are mitochondrially targeted or secreted (supplementary file S2, Supplementary Material online). These results agree with the experimental evidence showing that the PNX precursor acts both as a neuropeptide precursor, containing the physiologically active PNX-14 and PNX-20 peptides (Yosten et al. 2013a;Cowan et al. 2015) and as a stabilizing chaperon of COX1, a subunit of the cytochrome-c complex in mitochondria (Mcilwraith and Belsham 2018).

### The Nesfatin-1 Precursor, but not the Peptide, Shows Conservation Beyond Metazoans

**Fig. 2.**
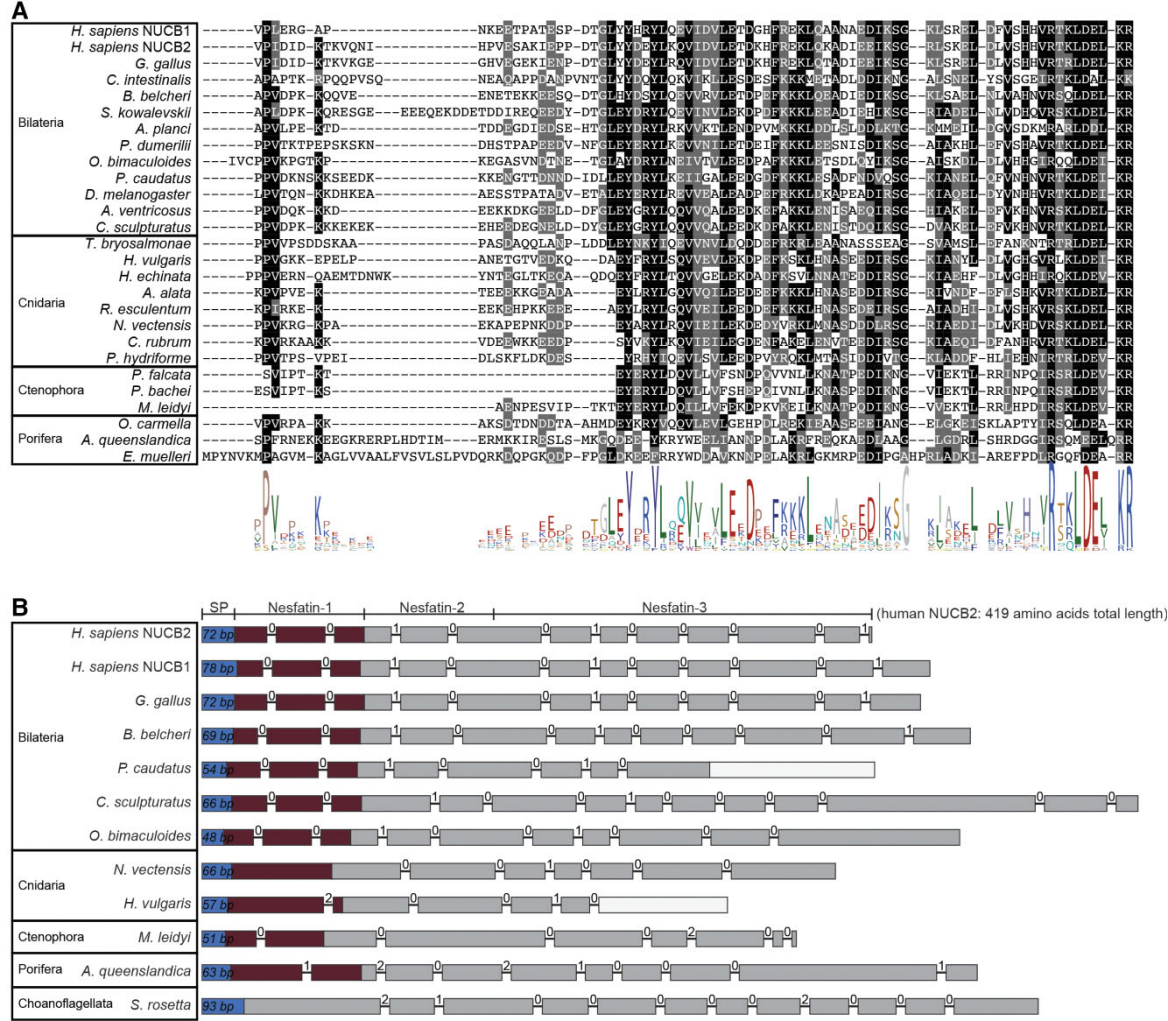
Sequence alignment of nesfatin-1 and genomic structure of NUCB precursors. (*A*) Alignment of the N-terminal NUCB precursor region containing the nesfatin-1 peptides. The conserved residues are highlighted, with conservation in more than 50% of sequences shown in black, and conservative substitutions shown in gray. (*B*) The genomic exon–intron structure of NUCB precursors. The regions encoding the signal peptides are shown in blue. The nesfatin-1-peptide coding region is indicated in dark red. Introns are shown as lines, with the phase of the introns shown above the lines. An empty/white box indicates a missing part in the mRNA-genome alignment.

We identified homologous NUCB precursors in bilaterian, cnidarian, placozoan, ctenophore, and poriferan species, as well as in choanoflagellates and *Tunicaraptor*. However, the N-terminal region that contains the mature neuropeptide nesfatin-1 is less conserved in sponges (fig. 2*A*) and not identifiable in *T. adhaerens*, choanoflagellates or *Tunicaraptor* (fig. 2*B*, supplementary figs. S2 and S3, Supplementary Material online). These sequences only show conservation in the C-terminal part of the precursor, with the strongest conservation in the region corresponding to the N-terminus of the human nesfatin-3 peptide, which contains an EF-hand domain (supplementary fig. S2, Supplementary Material online). This partial conservation suggests a premetazoan origin of the NUCB precursor protein that later evolved the nesfatin-1 peptide in the ancestral metazoan lineage, with a potential secondary loss of the peptide region in placozoans. In contrast to the PNX precursor gene, the gene structure of the NUCB gene is not widely conserved and differs already between deuterostome and protostome species in the region encoding the C-terminal part of the precursor (fig. 2*B*). The only resemblance in gene structure across bilaterian sequences is that the region encoding the nesfatin-1 peptide is divided by two phase-0 introns. This feature is not found in any nonbilaterian species, except in ctenophores in which a phase-0 intron is present in the region that matches the first phase-0 intron of bilaterians. Thus, the homology of NUCB precursors from bilaterians, nonbilaterian metazoans, choanoflagellates, and *Tunicaraptor* was primarily established through sequence-similarity, reciprocal blast, and alignment of the entire precursors. The *T. adhaerens*, choanoflagellate, and *Tunicaraptor* NUCB sequences show similarity in their C-terminal part but not in the N-terminal region of the precursor that encodes the nesfatin-1 peptide in the other species. This suggests that the nesfatin-1 peptide is not present in placozoans, choanoflagellates, or *Tunicaraptor* (supplementary figs. S2 and S3, Supplementary Material online).

### GPR173 is Unlikely to be a PNX Receptor

The SREB family of receptors was named after their expression in the central nervous system and a high level of conservation between vertebrate species (Matsumoto et al. 2000; Breton et al. 2021). There are at least three SREB receptors in vertebrates: SREB1 (GPR27), SREB2 (GPR85), and SREB3 (GPR173) (Matsumoto et al. 2000). These receptors are orphans, as no ligand–receptor assay has so far identified a potent ligand for them. Based on a “Deductive Reasoning Strategy”, SREB3 has been proposed as a potential receptor for the neuropeptide PNX (Stein et al. 2016). Some in vivo experiments further indicate, although indirectly, a ligand–receptor relationship between PNX and SREB3/GPR173. In female rats, exogenously administered PNX induces a preovulatory-like secretion of luteinizing hormone (LH). When GPR173 expression was reduced by siRNA treatment, this effect of PNX on LH secretion was significantly reduced (Stein et al. 2016). Furthermore, the siRNA knockdown of GPR173 doubled the length of the estrous cycle in female rats (Stein et al. 2016) similar to the knockdown of PNX that increased the estrous cycle by >50% (Yosten et al. 2013a).

To further explore whether SREBs are PNX receptors, we searched for homologs of SREB receptors across animals. An initial cluster-based analysis showed that the SREB receptors form a tight cluster, indicating high levels of conservation (supplementary fig. S4, Supplementary Material online), as previously shown (Matsumoto et al. 2000). The only connection of SREBs to any other GPCR cluster is to monoaminergic receptors (at an *e*-value of 1*e*−27). Therefore, we used the SREB cluster with monoamine receptors as outgroups to carry out a phylogenetic analysis.

SREB receptors have been described as vertebrate-specific, as they have not been identified in nonvertebrate chordates or in invertebrates in previous studies (Matsumoto et al. 2000; Breton et al. 2021). By searching an expanded group of species, we found that the SREB receptors are present in one copy in several invertebrates, including cephalochordates, ambulacrarians, ecdysozoans, and lophotrochozoans (supplementary fig. S5, Supplementary Material online). This demonstrates that SREB receptors are of urbilaterian origin, and the three copies of SREBs present in vertebrates are consistent with the hypothesis suggesting two rounds of whole-genome duplication in vertebrates (Holland et al. 1994; Dehal and Boore 2005; Blomme et al. 2006), with one of the copies potentially lost.

We could not identify SREB receptors in any of the nonbilaterian species, contrasting with the much broader phyletic distribution of the PNX precursors. This indicates that PNX peptides in these organisms must signal via other types of receptors. The phyletic mismatch between PNX and SREB also casts doubt on the suggested ligand–receptor relationship between human PNX and SREB3. Most other GPCR families show tight co-occurrence with their peptide ligands across taxa (Jékely 2013; Mirabeau and Joly 2013).

**Fig. 3.**
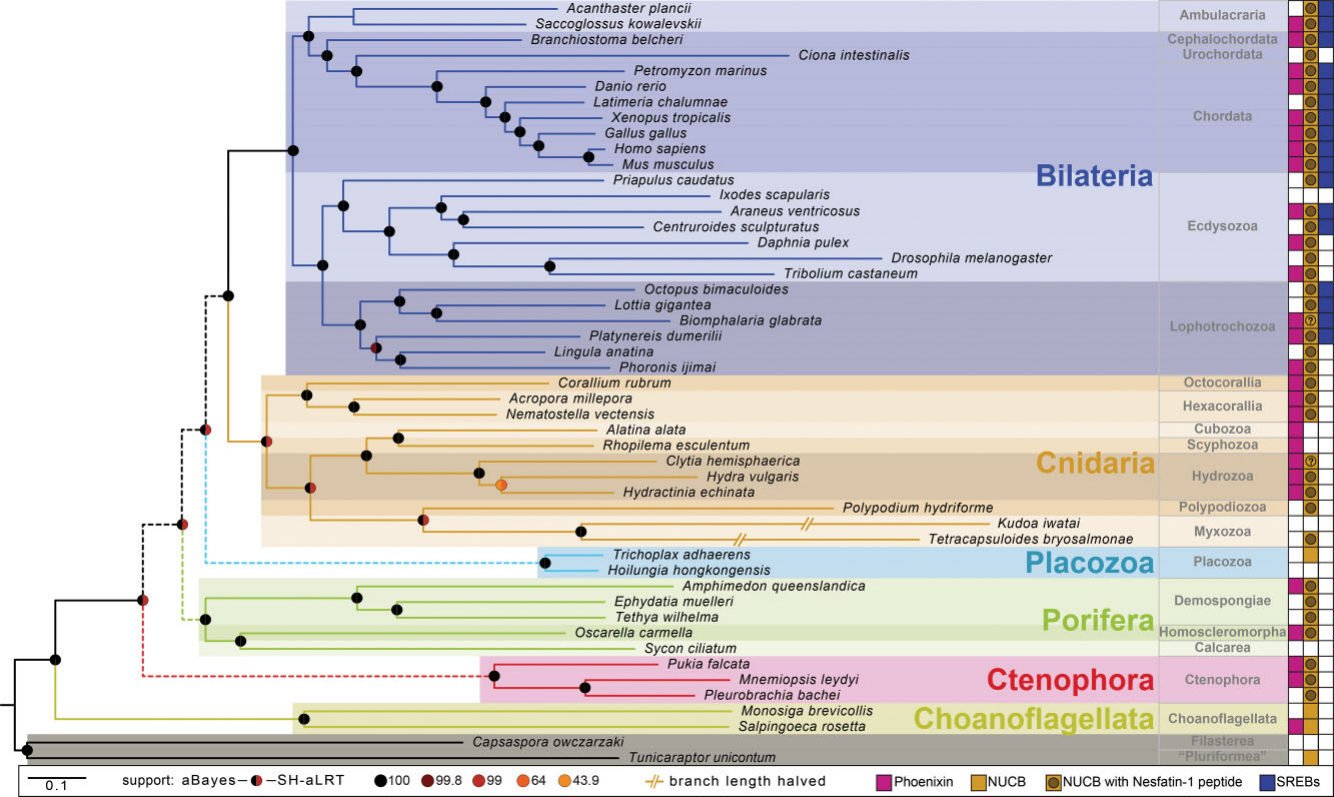
Presence and absence of PNX and nesfatin precursors and SREB receptors in the investigated species. Phylogenomic tree of the investigated species, annotated with the presence/absence of PNX precursor, NUCB and its nesfatin-1 peptide, and SREB receptors. The PNX neuropeptide precursor is conserved across metazoans and in the choanoflagellate *S. rosetta*, whereas GPR173 (proposed as a potential receptor for this peptide) is only present in Bilateria. The NUCB precursor gene is conserved across metazoans and present in choanoflagellates and *Tunicaraptor*, whereas the nesfatin-1 peptide is only encoded in metazoans. A question mark indicates that the NUCB sequence was only partially recovered with the N-terminal part that encodes the nesfatin-1 peptide missing in the transcriptome. An unfilled box indicates that the corresponding gene was not identified. Dashed lines in main bilaterian branches indicate generally contradicting results in different phylogenomic analyses.

To directly test if the human PNX-14 peptide is able to activate any of the three human SREB receptors, next we carried out calcium mobilization assays. We used two different promiscuous chimeric G-proteins, Gqi9 and Gqs5 separately, to test for coupling to different G-alpha subunits. We could not detect any activation of the three SREB receptors by the PNX peptide, even at very high peptide concentrations (up to 1*e*−4M; supplementary fig. S6, Supplementary Material online). In the same assay, we could get reliable activation of two other GPCRs by their cognate peptide ligand. It has to be noted that these types of deorphanization assays may not work for all ligand–receptor pairs (Foster et al. 2019; Hauser et al. 2020). Nevertheless, the nonmatching evolutionary pattern between PNX and SREB (fig. 3) together with the negative receptor activation assay (supplementary fig. S6, Supplementary Material online) suggests that this receptor–ligand pairing may not be correct and should be re-evaluated. Finally, the PNX–GPR173 pairing is not the only case in which the experimental ligand–receptor assays are not consistent with the proposed pairing obtained by the use of the “Deductive Reasoning Strategy.” In 2013, the proinsulin Connecting Peptide (C-Peptide), was proposed as the ligand for the receptor GPR146 using this methodology ([Bibr msac051-B93][Bibr msac051-B96]). Recently, using mass redistribution and β-arrestin–based ligand–receptor assays, it was shown that the proinsulin C-peptide does not activate GPR146 (Lindfors et al. 2020).

## Conclusions

Our bioinformatic survey identified PNX and nesfatin as ancient neuropeptides with premetazoan origin. To our knowledge, these are the first neuropeptides to be identified in sponges and with broad conservation across animals, including sponges, ctenophores, cnidarians, and bilaterians. The presence of a PNX peptide in choanoflagellates demonstrates that some animal neuropeptides have premetazoan origin and predate nervous systems. Many other neuronal molecules, including neurosecretory components (Göhde et al. 2021), postsynaptic proteins (Burkhardt et al. 2014), and voltage-gated channel subunits (Moran and Zakon 2014), have a similar history, suggesting the stepwise assembly of the synaptic and neurosecretory machinery from pre-existing components at the origin of neurons (Arendt 2020; Burkhardt and Jékely 2021).

What could be the functions of NUCB and PNX in choanoflagellates and non-neuronal sponges? The precursor sequences indicate that both are secreted proteins, and PNX is processed to release a mature PNX peptide in both choanoflagellates and sponges. An interesting possibility is that these proteins regulate feeding. In mammals, nesfatin-1 produces anorexigenic effects, whereas PNX promotes feeding and drinking behavior (Maejima et al. 2009; Stengel et al. 2012; Schalla et al. 2017). Uncovering similar potential functions in nonbilaterians will require functional studies.

Another interesting avenue for future research is the study of the subcellular localization of the PNX precursors in nonbilaterians. While the PNX precursor in mammals releases mature PNX neuropeptides, the precursor also has mitochondrial localization and function. According to our analyses, the majority of PNX precursors are predicted to be both secreted and mitochondrially localized. This ambiguity suggests that PNX has a conserved moonlighting function across many species.

Given the ancestral origins, similar distributions and complementary roles of PNX and nesfatin-1 in some physiological functions in vertebrates, we speculate that these two peptides may have functionally coevolved since metazoan origins.

Nesfatin-1 and PNX also broadly coexpress in the rat hypothalamus, with over 70% of the PNX-expressing neurons coexpressing nesfatin-1 (Pałasz et al. 2015). Besides their antagonistic effects on feeding, the two peptides also have opposing roles in the regulation of anxiety and fear-like behavior. PNX has an anxiolytic effect in mice (Jiang et al. 2015) and likely also humans (Hofmann et al. 2017), whereas nesfatin-1 increases anxiety (Merali et al. 2008; Hofmann et al. 2015). In addition, PNX administration leads to increased nesfatin-1-immunoreactivity in rats (Friedrich et al. 2019), indicating a functional interplay.

Overall, our findings suggest that secretion and intercellular signaling by peptides in animal evolution evolved before neurons and synapses, in agreement with the “chemical brain” theory for the origin of nervous systems (Jékely 2021). In future, it will be interesting to test if the two peptides coexpress and have antagonistic functions also in different invertebrate nervous systems. Equally exciting will be to explore the function of these precursors in sponges and choanoflagellates. Will the peptides make these organisms anxious or hungry?

## Materials and Methods

### Transcriptomic Resources

To identify the phyla to be included for the analysis of PNX and nesfatin-1 precursors, we performed an initial BlastP analysis in the NCBI database including metazoa, choanozoa, plants, fungi, and prokaryotes. The PNX precursor (SMIM20) from human and *Nephrops norvegicus* (Yosten et al. 2013a; Nguyen et al. 2018) and the nesfatin-1 precursor from human and *Drosophila melanogaster* were used as queries. No candidates were identified in plants and fungi. Thus, transcriptomes from different clades of metazoans, choanoflagellates, a filasterean, and the flagellate *Tunicaraptor unikontum* were obtained from different public databases (see supplementary file S1, Supplementary Material online). We translated the transcripts into protein sequences with TransDecoder (TransDecoder; http://transdecoder.github.io/) with a minimum length of 50 amino acids. To assess the completeness of the transcriptomes, we ran BUSCO v5.2.1 (Manni et al. 2021) in protein mode and with the lineage set to “eukaryote” with the database “eukaryota_odb10” (Creation date of the database: September 2021, number of BUSCOs: 255).

### Phylogenomic Analysis

To build a tree representing the relationships of the 49 species studied, we carried out a phylogenomic analysis with the output of the BUSCO analysis. BUSCO data sets comprise genes evolving under “single-copy control” (Waterhouse et al. 2011) and are near-universally present as single-copy orthologs across lineages. The eukaryotic database has 255 single-copy orthologs. We aligned these orthologs from each species individually with MAFFT v7 using the iterative refinement method L-INS-i (Katoh et al. 2002). The alignment was trimmed with the TrimAl software using the gappy-out method (Capella-Gutiérrez et al. 2009). Then, we concatenated the trimmed alignments with FASconcatG (Kück and Longo 2014) to assemble a concatenated supermatrix of 114,163 amino acid positions (supplementary file S3, Supplementary Material online). To build a species tree, we used IQ-TREE2 with the maximum-likelihood method under the LG + G4 model (Nguyen et al. 2015). The tree was rooted in the filasterean + *Tunicaraptor* clades. It is important to note that this phylogenomic analysis does not account for compositional bias and has been run with a homogeneous model (LG) only. The tree is merely used as a guide to map the evolutionary pattern of PNX, nesfatin-1, and the GPR173 across the species tree.

### PNX and Nesfatin-1 Precursor Identification and Alignment

We identified the PNX precursor sequences by using the PNX precursor (SMIM20) from human and *Nephrops norvegicus* (Yosten et al. 2013a; Nguyen et al. 2018) as queries. To search for nesfatin-1 precursor sequences, we used the human and *Drosophila melanogaster* precursors as queries (Zandawala et al. 2017). We used a BlastP search with an *e*-value of 1*e*−2 as the threshold to collect homologous sequences. To minimize the possibility of false positives, we manually curated the sequence list. After testing different signal peptide prediction (SignalP 3.0, SignalP 4.1, SignalP 5.0, SignalP 6.0) and subcellular localization tools (DeepLoc 1.0, TargetP 2.0) with the human PNX precursor, we decided to use signalP-3.0 to detect signal peptides. The initially detected precursors were then used as new query sequences in a second BlastP search to detect potentially hidden orthologs. To align the full-length precursors and predicted mature peptides derived from them, we used MUSCLE (Edgar 2004). The lists of the sequences used for these alignments are available in supplementary files S4 and S6, Supplementary Material online.

### Gene-Structure Analyses of PNX and Nesfatin-1 Precursor Sequences

In all the species in which we identified PNX and/or nefastin-1 precursors, we also searched for the corresponding genes with Blast in the GenBank database. For gene-structure analysis, we selected at least one species from each of the major clades of metazoans and the choanoflagellate *S. rosetta* and we retrieved the transcripts and genomic regions. We used the tool Splign (Kapustin et al. 2008) to determine the exon/intron structure of the genes (https://www.ncbi.nlm.nih.gov/sutils/splign/splign.cgi). Based on these data, the gene-structure diagrams were drawn in Adobe Illustrator CS6. The output of the Splign analysis is available in supplementary files S5 and S7, Supplementary Material online (for PNX and nesfatin-1, respectively).

### GPR173 Identification and Phylogenetic Analysis

To identify GPR173 receptors, we obtained a database of vertebrate SREB sequences, including GPR173, GPR85 and GPR27 from (Breton et al. 2021). From these sequences, we produced a Hidden Markov Model (HMM) and used this to mine the 49 species investigated. HMM models were run in HMMR3 with an *e*-value of 1*e*−15. The same SREB sequences were used to carry out similarity-based searches using BlastP with an *e*-value cutoff of 1*e*−15. We merged these two databases and ran CD-Hit (Fu et al. 2012) to eliminate redundant sequences (at a 99% threshold). To identify the sequences that are closely related to the GPR173 sequences, we ran a cluster-based analysis in CLANS (Frickey and Lupas 2004).The CLANS analysis is available as supplementary fig. S4, Supplementary Material online. To identify clusters, we used the convex-clustering option with 100 jack-knife replicates. The SREB receptors are extremely well conserved and form an easily recognizable cluster. To analyze the phylogeny of SREB receptors, the cluster containing these receptors together with monoaminergic receptors were parsed and used for tree building. We aligned the sequences with MAFFT version 7, with the iterative refinement method E-INS-i. Alignments were trimmed with TrimAl in gappy-out mode (Capella-Gutiérrez et al. 2009). To calculate maximum-likelihood trees, we used IQ-tree2 with the LG + G4 model. To calculate branch support, we ran 1,000 replicates with the aLRT-SH-like and aBayes methods (Minh et al. 2020). The sequences used for the phylogenetic analysis are available in supplementary S8, Supplementary Material online, the trimmed alignment is available in supplementary S9, Supplementary Material online. The raw nexus tree of the SREB receptors is available in supplementary file S10, Supplementary Material online.

### GPR173 Deorphanisation Assays

We ordered the synthetic mature peptide PNX14 from GenScript with a purity of >95%. The receptors GPR173, GPR85, and GPR27 were purchased from the GenScript GenEZ human open reading frame (ORFs) database (Accession Nos. NM_018969.6, NM_001146266.1, and NM_018971.2, respectively) and cloned into a pcDNA3.1(+) vector with EcoRV enzyme and a blunt cloning strategy. We expressed the receptors in HEK293 cells that were stably expressing the calcium-sensitive bioluminescent reporter green fluorescent protein (GFP)-aequorin fusion protein (G5A). This cell line was purchased from Angio-proteomie (CAT no. cAP-0200GFP-AEQ-Cyto). The HEK293-G5a cells were maintained at 37 °C in an 5% CO_2_ atmosphere, in 96-well plates containing 100 µl of Dulbecco's modified eagle medium (DMEM) high-glucose glutamax medium (Thermo; Cat. No. 10566016) supplemented with 10% fetal bovine serum (Thermo; Cat. No. 10082147). Upon reaching confluency of ∼85%, we transfected the cells with the plasmid containing the receptor to be tested and a plasmid containing the promiscuous Gαqi9 (Addgene; Cat. No. 125711 [Masharina et al. 2012]) or Gαqs5 (Addgene; Cat. No. 24498) (Conklin et al. 1996).

Transfections were carried out with 60 ng of each plasmid and 0.35 µl of the transfection reagent Transfectamine 5000 (AAT-bioquest; Cat. No. 60022). Two days post-transfection, we removed the culture medium and substituted it for fresh DMEM-medium supplemented with 4 mM coelenterazine-H (Thermo Fisher Scientific; Cat. No. C6780). After an incubation period of 3 h, we exposed the cells to synthetic PNX-14 peptide diluted in DMEM-medium in concentrations ranging from 10^−4^ to 10^−6^ M. Luminescence levels were recorded over a 60 s period in a FlexStation 3 Multi-Mode Microplate Reader (Molecular Devices).

We integrated the luminescence data over a 60 s measurement period. A minimum of two independent transfections with triplicate measurements were made for each concentration, and the average of each was used to normalize the responses. We normalized the responses to the maximum response obtained by the addition of 100 µM ATP in each experiment (100% activation) and to the response obtained with the vehicle media (0% activation). As positive control for the Gαqi/9 protein, we used the *Clytia hemisphaerica* MIH receptor and one of its MIH-peptide ligands, RPRYamide (Quiroga Artigas et al. 2020). As positive control for the Gαqs5 protein, we used the human serotonin receptor 4 (5-HTR4) purchased from the GenScript GenEZ human ORFs database (Accession No. NM_000870.6) and tested it with serotonin hydrochloride purchased from Sigma-Aldrich (Cat No. H9523). For the positive controls, responses were normalized to the maximum response obtained by the addition of the activating compound (i.e., serotonin or RPRYamide peptide), dose–response curves were fitted with a four-parameter curve based on the normalized data from the average of three independent transfections using Prism 8 (GraphPad, La Jolla, CA, USA). The raw data obtained from the deorphanization assays shown in supplementary fig. S6, Supplementary Material online are available in supplementary file S11, Supplementary Material online.

## Supplementary Material

msac051_Supplementary_Data
