# Supplementary material for: Premetazoan Origin of Neuropeptide Signaling
Source: Mol Biol Evol. 2022 Mar 12;39(4):msac051. doi: 10.1093/molbev/msac051 (PMC9004410; doi:10.1093/molbev/msac051)

Species/Software	SignalP3.0	SignalP6.0	DeepLoc	TargetP2.0
**Bilateria**				
Homo sapiens	Signal peptide	No Signal peptide	Signal peptide/Secretory pathway	Mitochondria/Membrane
Mus musculus	Signal peptide	No Signal peptide	Signal peptide/Secretory pathway	Mitochondria/Membrane
Gallus gallus	Signal peptide	No Signal peptide	Signal peptide/Secretory pathway	Mitochondria/Membrane
Xenopus tropicalis	Signal peptide	No Signal peptide	Signal peptide/Secretory pathway	Mitochondria/Membrane
Danio rerio	Signal peptide	No Signal peptide	Signal peptide/Secretory pathway	Mitochondria/Membrane
Petromyzon marinus	Signal peptide	No Signal peptide	Signal peptide/Secretory pathway	Mitochondria/Membrane
Branchiostoma belcheri	Signal peptide (dicp HMM/NN)	No Signal peptide	Other	Mitochondria/Membrane
Saccoglossus kowalevskii	Signal peptide (dicp HMM/NN)	No Signal peptide	Signal peptide/Secretory pathway	Mitochondria/Membrane
Platynereis dumerilii	Signal peptide (dicp HMM/NN)	No Signal peptide	Signal peptide/Secretory pathway	Mitochondria/Membrane
Daphnia pulex	Signal peptide (dicp HMM/NN)	No Signal peptide	Signal peptide/Secretory pathway	Mitochondria/Membrane
Tribolium castaneum	Signal peptide (dicp HMM/NN)	No Signal peptide	Signal peptide/Secretory pathway	Mitochondria/Membrane
Araneus ventricosus	Signal peptide (dicp HMM/NN)	No Signal peptide	Other	Mitochondria/Membrane
Phoronis ijimai	Signal peptide	No Signal peptide	Other	Mitochondria/Membrane
Biomphalara glabrata	Signal peptide	No Signal peptide	Signal peptide/Secretory pathway	Mitochondria/Membrane
**Cnidaria**				
Acropora millepora	Signal peptide	No Signal peptide	Signal peptide/Secretory pathway	Mitochondria/Membrane
Nematostella vectensis	Signal peptide	No Signal peptide	Signal peptide/Secretory pathway	Mitochondria/Membrane
Corallium rubrum	Signal peptide	No Signal peptide	Signal peptide/Secretory pathway	Mitochondria/Membrane
Clytia hemisphaerica	Signal peptide	No Signal peptide	Signal peptide/Secretory pathway	Mitochondria/Membrane
Hydra vulgaris	Signal peptide	No Signal peptide	Signal peptide/Secretory pathway	Mitochondria/Membrane
Hydractina echinata	Signal peptide	No Signal peptide	Signal peptide/Secretory pathway	Mitochondria/Membrane
Rhopilema esculentum	Signal peptide (dicp HMM/NN)	No Signal peptide	Signal peptide/Secretory pathway	Mitochondria/Membrane
Alatina alata	Signal peptide (dicp HMM/NN)	No Signal peptide	Other	Mitochondria/Membrane
**Ctenophora**				
Mnemiopsis leidyi	Signal peptide	No Signal peptide	Other	Mitochondria/Membrane
Pukia falcata	Signal peptide	No Signal peptide	Signal peptide/Secretory pathway	Mitochondria/Membrane
**Porifera**				
Oscarella carmela	Signal peptide (dicp HMM/NN)	No Signal peptide	Signal peptide/Secretory pathway	Mitochondria/Membrane
Amphimedon queenslandica	Signal peptide (dicp HMM/NN)	No Signal peptide	Other	Mitochondria/Membrane
**Choanoflagellates**				
Salpingoeca rosetta	Signal peptide (dicp HMM/NN)	No Signal peptide	Signal peptide/Secretory pathway	Mitochondria/Membrane

HMM = Hidden Markov Model; NN = Neural network; dicp HMM/NN = discrepancy in cleavage position between HMM and NN prediction

Prediction of signal peptide and subcellular localization for the human phoenixin precursor SMIM20 using different online software.

>NP_001138904.1 small integral membrane protein 20 [Homo sapiens]
MSRNLRTALIFGGFISLIGAAFYPIYFRPLMRLEEYKKEQAINRAGIVQEDVQPPGLK
VWSDPFGRK

SignalP 3.0:

https://services.healthtech.dtu.dk/service.php?SignalP-3.0

SignalP-HMM result:

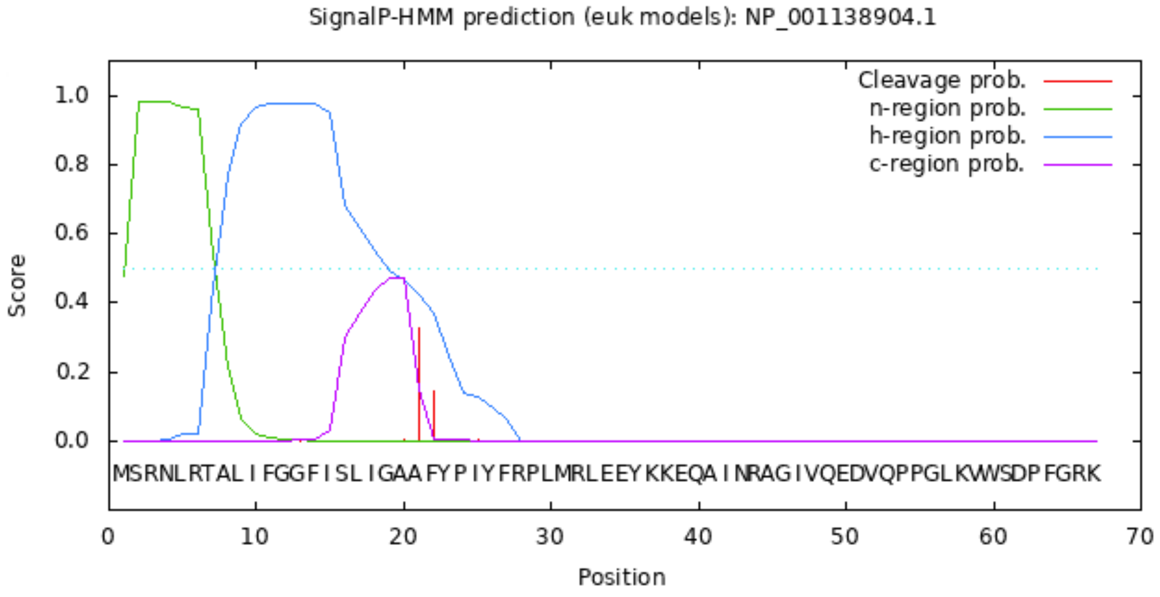

data

```
>NP_001138904.1
Prediction: Signal anchor
Signal peptide probability: 0.476
Signal anchor probability: 0.509
Max cleavage site probability: 0.324 between pos. 20 and 21
```

SignalP-NN result:

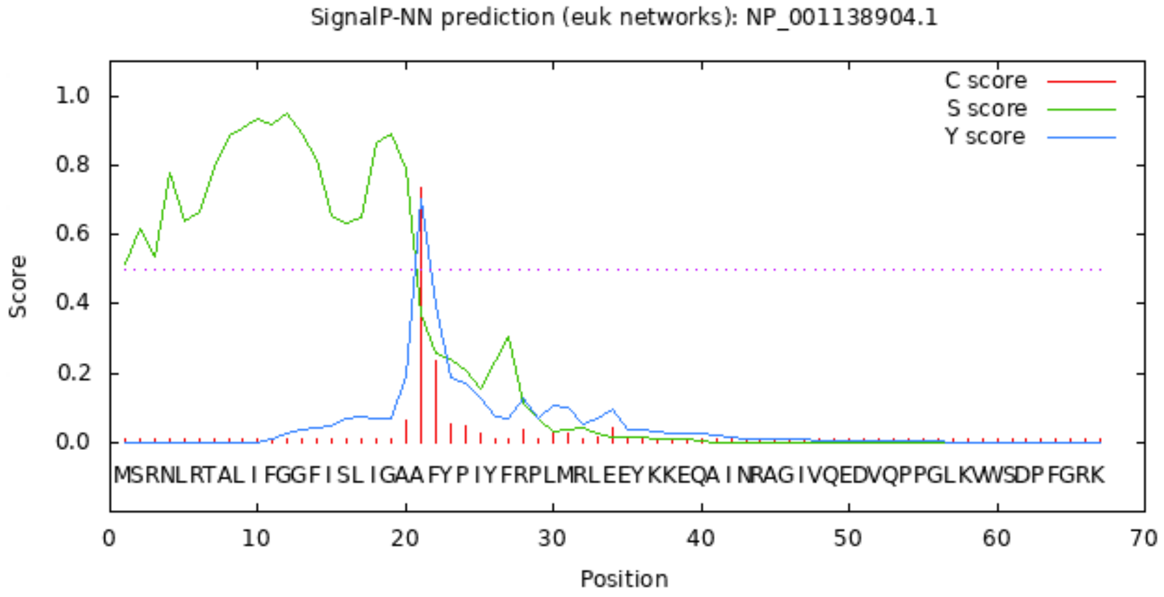

data

```
>NP_001138904.1        length = 67
# Measure  Position  Value  Cutoff  signal peptide?
  max. C     21       0.735   0.32   YES
  max. Y     21       0.705   0.33   YES
  max. S     12       0.949   0.87   YES
  mean S      1-20    0.765   0.48   YES
       D      1-20    0.735   0.43   YES
# Most likely cleavage site between pos. 20 and 21: IGA-AF
```

SignalP 4.1:

https://services.healthtech.dtu.dk/service.php?SignalP-4.1

```
# SignalP-4.1 euk predictions
>NP_001138904.1  small integral membrane protein 20 _Homo sapiens_
```

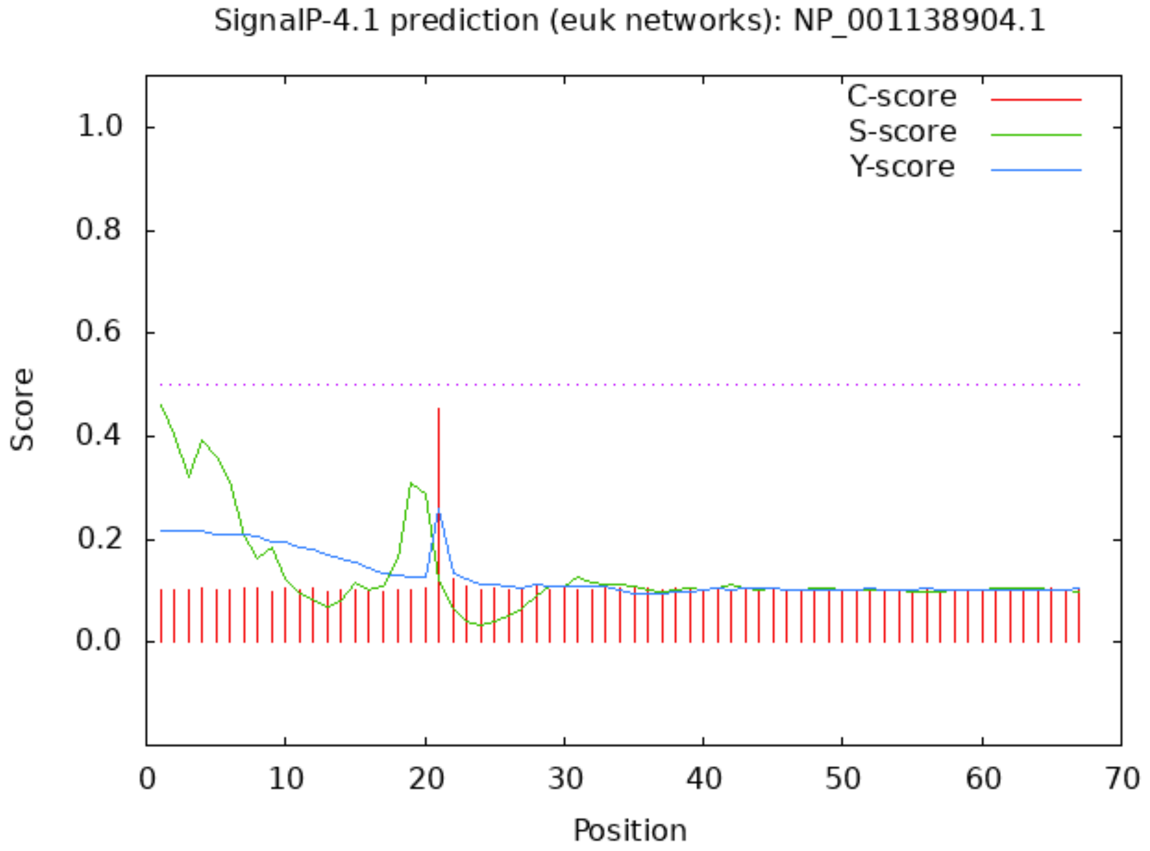


```
# Measure  Position  Value    Cutoff   signal peptide?
  max. C     21       0.452
  max. Y     21       0.260
  max. S      1       0.460
  mean S      1-20    0.217
       D      1-20    0.242   0.500    NO
Name=NP_001138904.1     SP='NO' D=0.242 D-cutoff=0.500 Networks=SignalP-TM
# data
# gnuplot script
```

SignalP 5.0:
https://services.healthtech.dtu.dk/service.php?SignalP-5.0

NP_001138904.1

Prediction: Other

Protein type	Signal Peptide (Sec/SPI)	Other
Likelihood	0.0849	0.9151

Download: PNG / EPS / Tabular

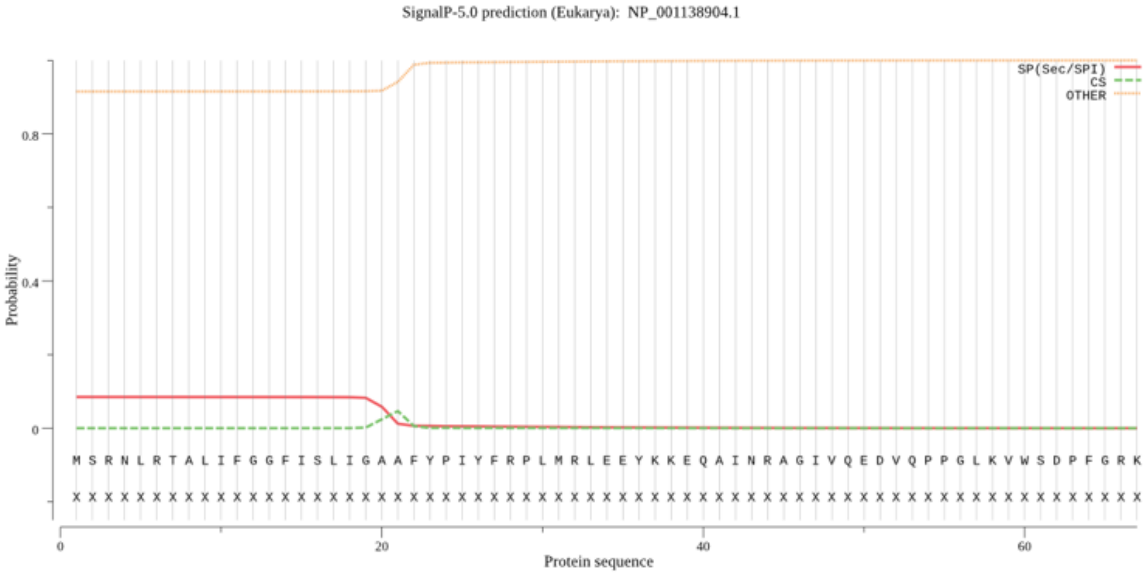

SignalP 6.0:

https://services.healthtech.dtu.dk/service.php?SignalP-6.0

NP_001138904.1
Prediction: Other

**Protein type**	**Other**	**Signal Peptide (Sec/SPI)**
**Likelihood**	1	0

Download: PNG / EPS / Tabular

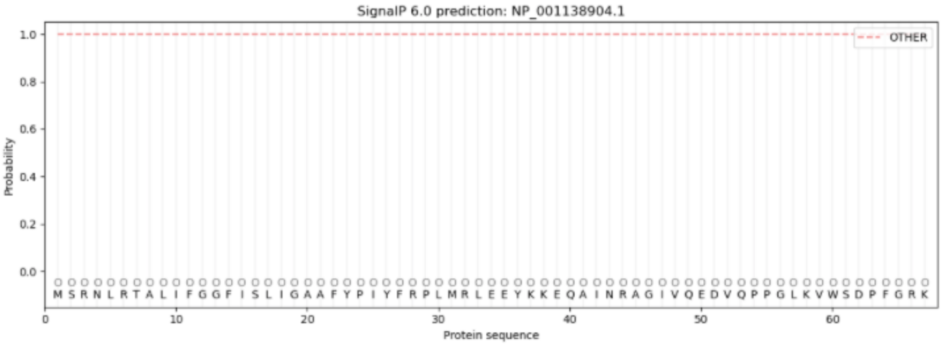

TargetP 2.0:

https://services.healthtech.dtu.dk/service.php?TargetP-2.0

NP_001138904.1
Prediction: Signal peptide

CS pos: 20-21. IGA-AF. Pr: 0.6258

Protein type	Other	Signal peptide	Mitochondrial transfer peptide
**Likelihood**	0.1951	0.7699	0.0351

Download: PNG / EPS / Tabular

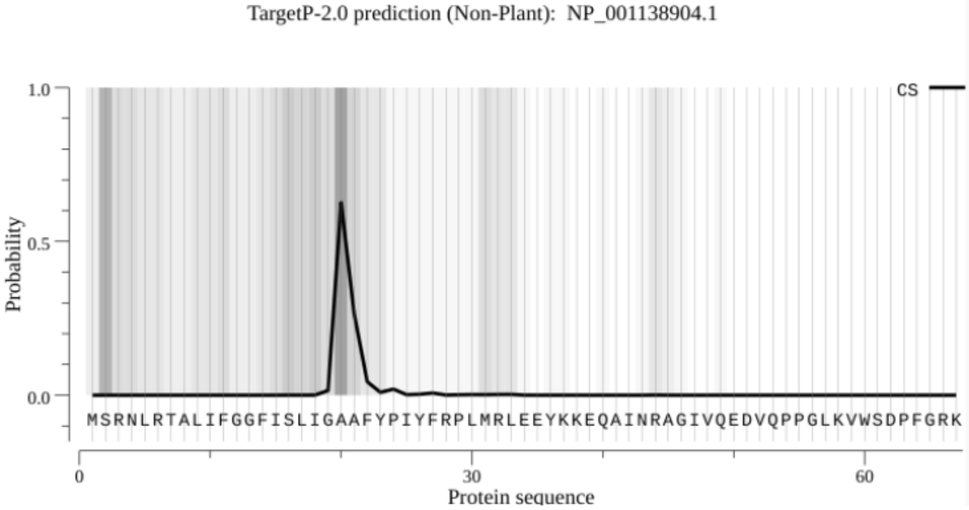

DeepLoc 1.0:

https://services.healthtech.dtu.dk/service.php?DeepLoc-1.0

Summary of 1 predicted sequences

Table of predicted subcelullar localizations. Use the help page for more detailed description of the output page.

Predicted proteins

NP_001138904.1

Prediction: Mitochondrion, Membrane

Localization	Mitochondrion	Endoplasmic reticulum	Plastid	Peroxisome	Nucleus	Lysosome/Vacuole	Golgi apparatus	Cell membrane	Cytoplasm	Extracell
Likelihood	0.9909	0.0042	0.003	0.0005	0.0003	0.0003	0.0003	0.0002	0.0001	0.0001

Type	Soluble	Membrane
Likelihood	0.002	0.998

Hierarchical Tree. Donwload: PNG / EPS

DeepLoc 1.0 Hierachical Tree:

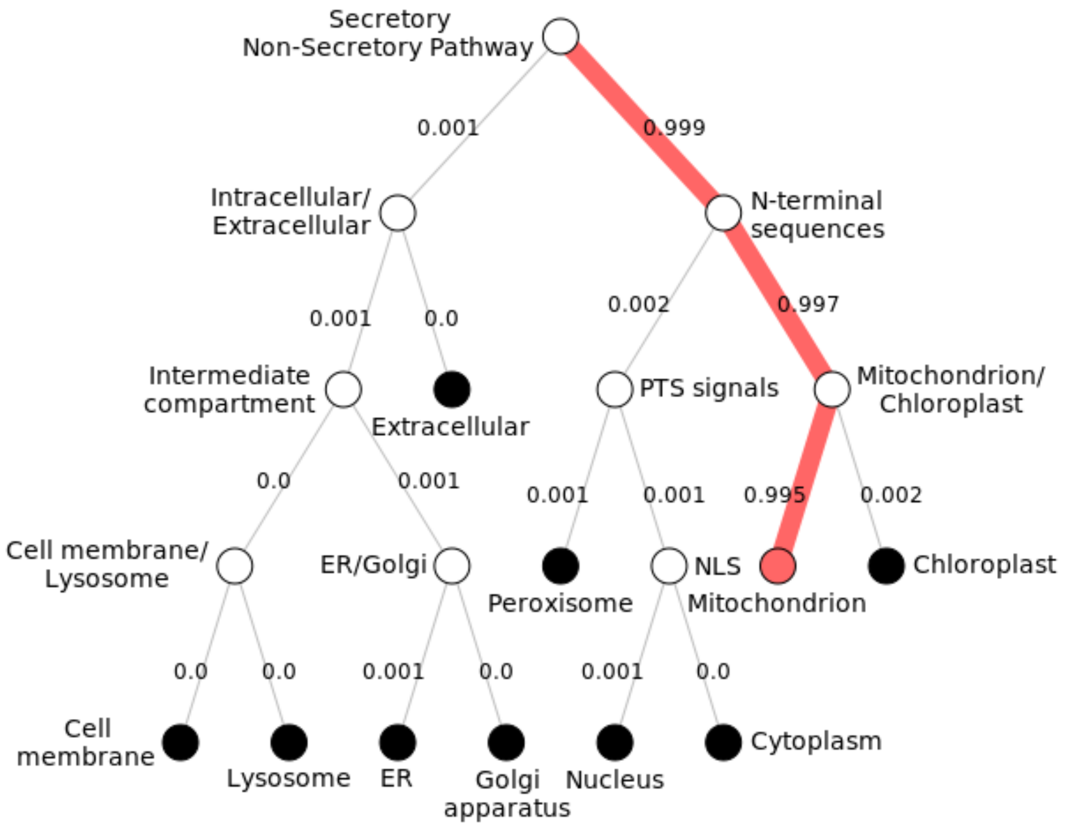

DeepLoc 1.0 Position Importance:

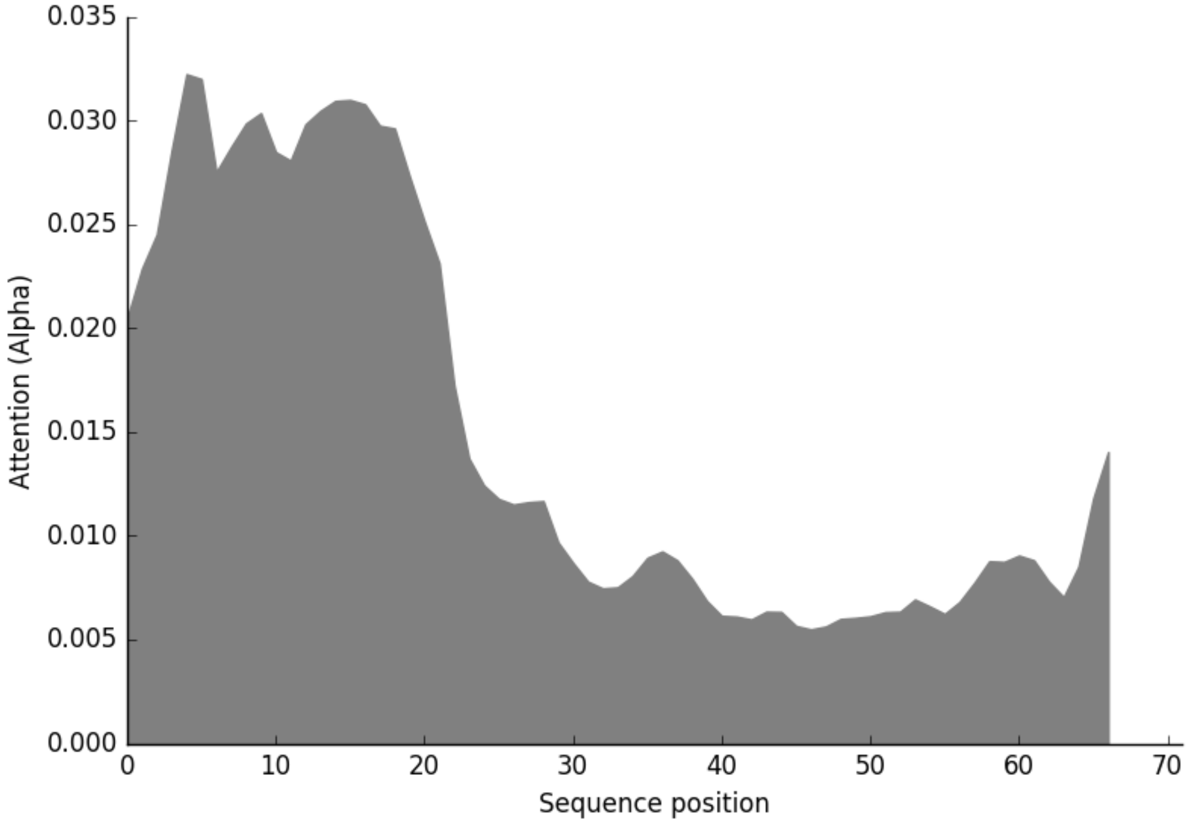

Supplement: msac051_Supplementary_Data [file msac051_supplementary_data.zip › Supplementary_File2_PhoenixinSPpredictions.pdf]
