# Supplementary material for: Premetazoan Origin of Neuropeptide Signaling
Source: Mol Biol Evol. 2022 Mar 12;39(4):msac051. doi: 10.1093/molbev/msac051 (PMC9004410; doi:10.1093/molbev/msac051)
Supplement: msac051_Supplementary_Data [file msac051_supplementary_data.zip › Supplementary_Materials.docx]

**Supplementary Figures**


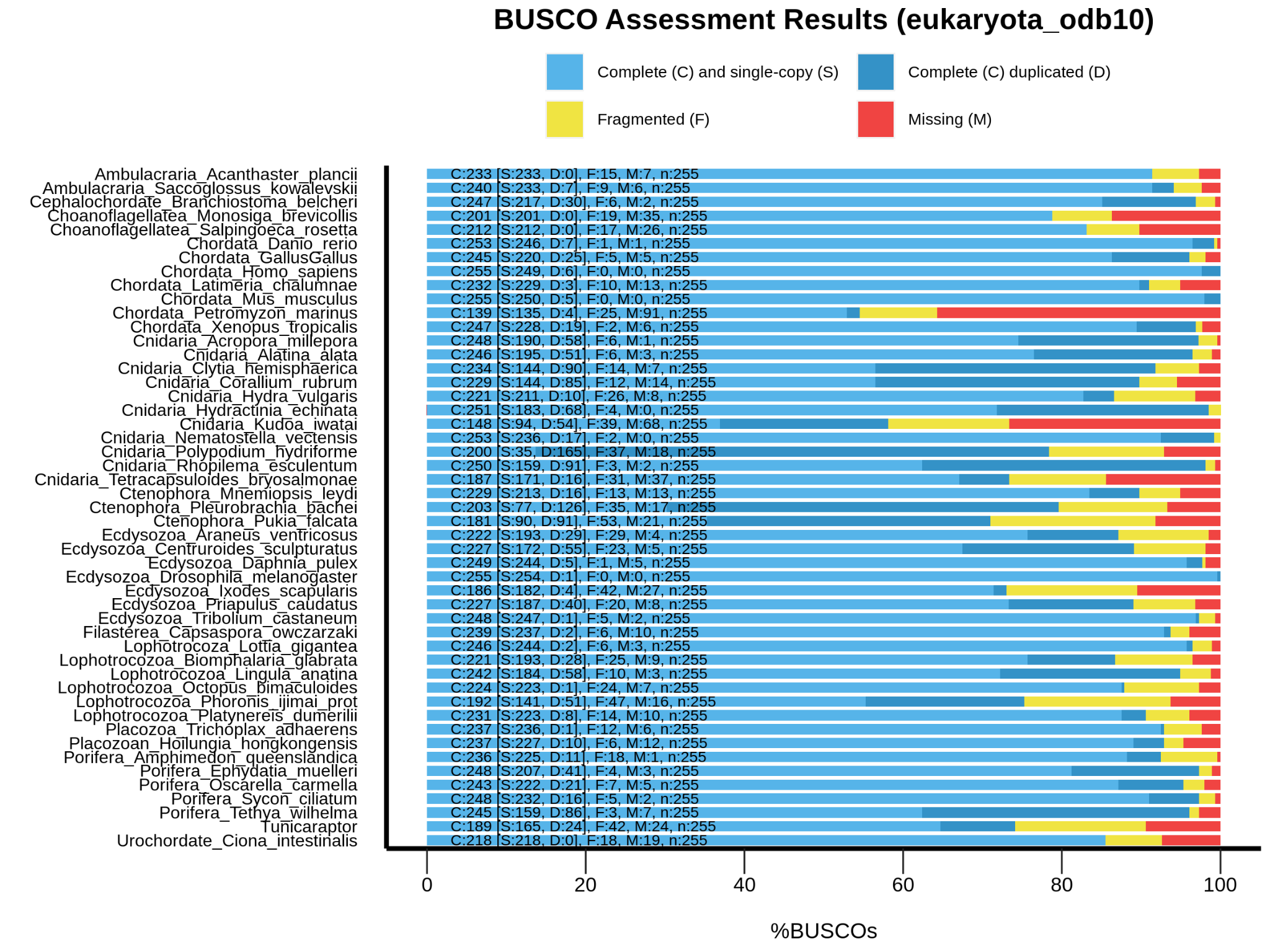


**Supplementary Figure 1. BUSCO completeness analysis.** Completeness of the transcriptomes used in the study.


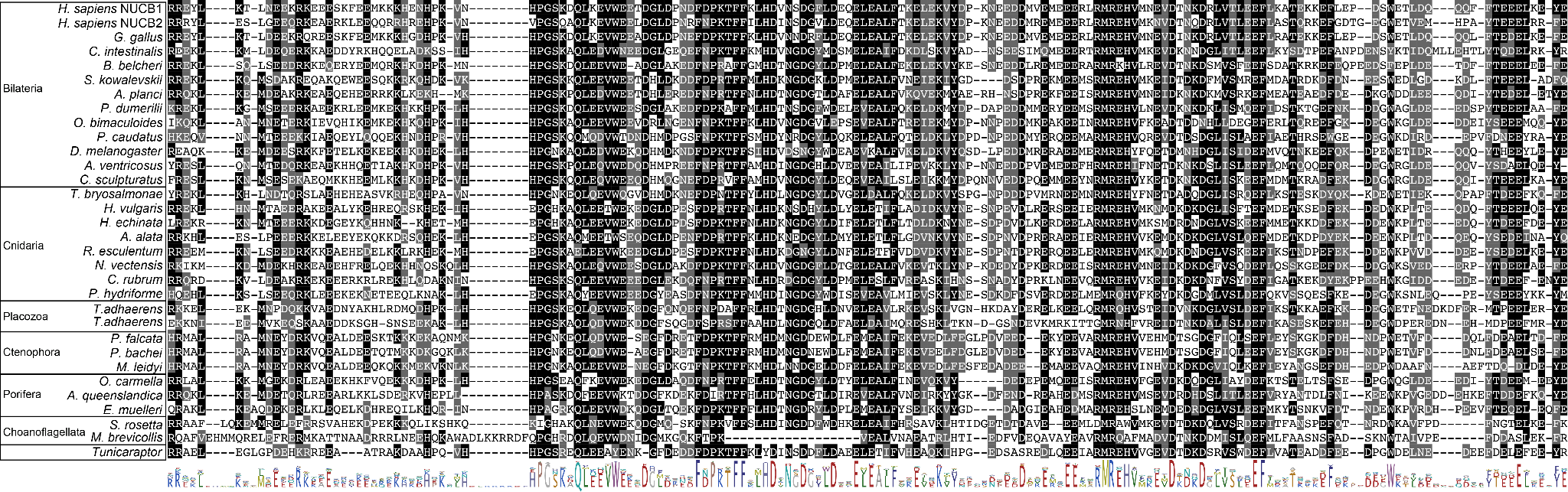


**Supplementary Figure 2. Alignment of the Nesfatin-3 region of NUCB precursors.** Alignment of the C-terminal region of the NUCB precursor corresponding to the nesfatin-3 region of the propeptides. The conserved residues are highlighted, with conservation in more than 50% of sequences shown in black and conservative substitutions shown in grey. A consensus sequence is shown below the alignment.

**Supplementary Figure 3. Whole-precursor alignment of NUCB precursors in all the species in which a homolog was identified**. The conserved residues are highlighted, with conservation in more than 50% of sequences shown in black and conservative substitutions shown in grey.


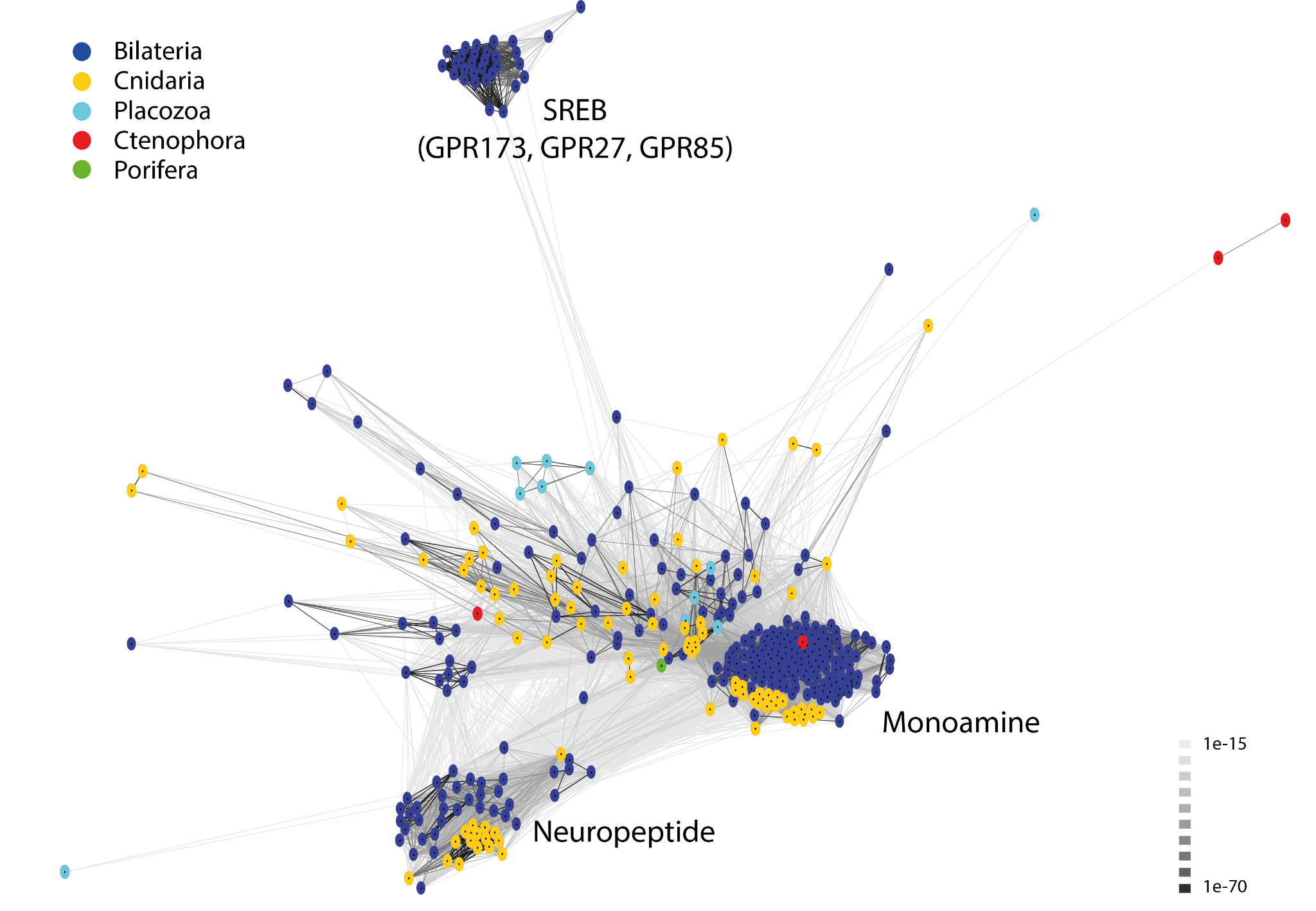


**Supplementary Figure 4. BLOSUM62 cluster map of metazoan SREB receptors and their closest GPCR relatives.** Nodes correspond to individual GPCR sequences and are coloured by taxon as indicated on the key. Edges correspond to BLAST connections of a P-value >1e-27. The cluster marked as SREB contains the GPR173, GPR27 and GPR85 homologs.


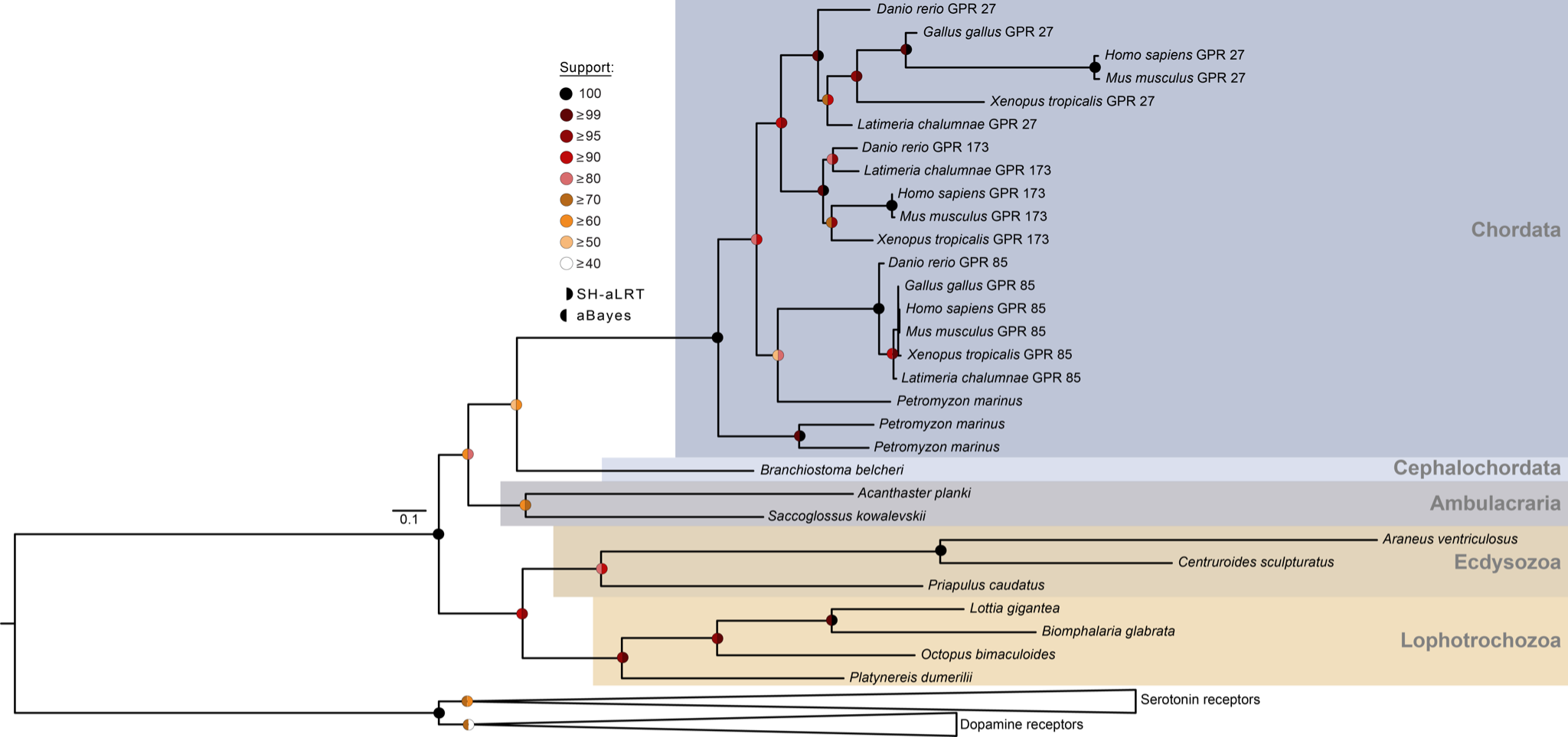


**Supplementary Figure 5. Phylogenetic tree showing the relationship of SREB receptors, including GPR173, GPR27 and GPR85**. SREB receptors were identified in cephalochordates, ambulacrarians, ecdysozoans and lophotrochozoans but they are absent in non-bilaterians. The circles represent branch support based on 1000 replicates as explained in the legend, and the coloured backgrounds represent different taxonomic groups, as shown in the key. Species names are as follows: Apla (*Acanthaster planci*), Aven (*Araneus ventricosus*), Bbel (*Branchiostoma belcheri*), Bgla (*Biomphalaria glabrata*), Csul (*Centruroides sculpturatus*), Drer (*Danio rerio)*, Ggal (*Gallus gallus*), Hsap (*Homo sapiens*), Lcha (*Latimeria chalumnae*), Lgig (*Lottia gigantea*), Mmus (*Mus musculus*), Obim (*Octopus bimaculoides*), Pcau (*Priapulus caudatus*), Pdum (*Platynereis dumerilii*), Pmar (*Petromyzon marinus*), Skow (*Saccoglossus kowalevskii*), Xtro (*Xenopus tropicalis*).


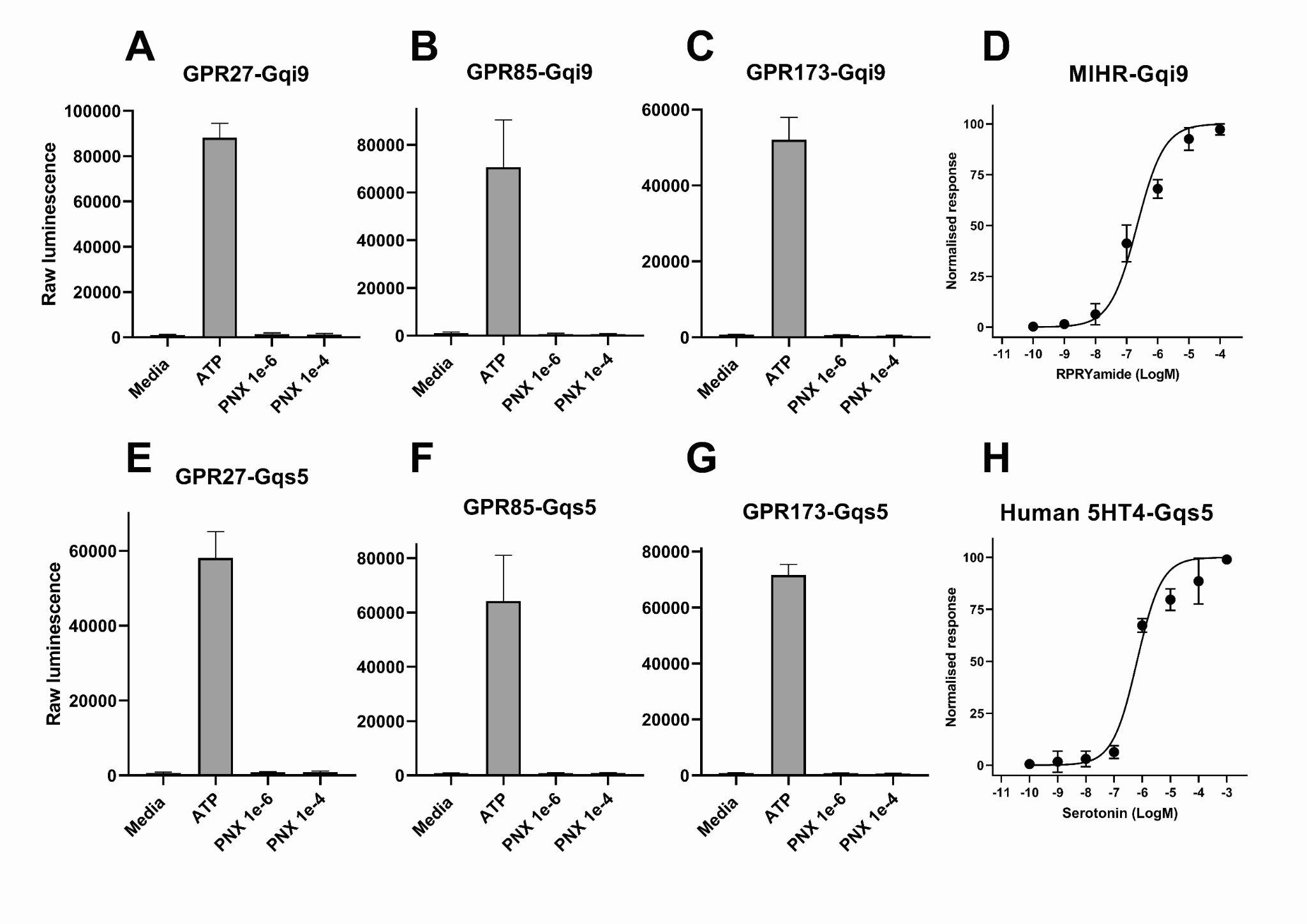


**Supplementary Figure 6. Receptor-ligand activation assay for human PNX-14 and human SREB receptors with different promiscuous G-proteins and positive controls.** (A) GPR27 with Gqi9. (B) GPR85 with Gqi9. (C) GPR173 with Gqi9. (D) Activation of the *Clytia hemisphaerica* MIH receptor by MIH peptide (positive control for Gqi9). (E) GPR27 with Gqs5. (F) GPR85 with Gqs5. (G) GPR173 with Gqi9. (H) Activation of the human serotonin receptor 4 by serotonin (positive control for Gqs5).

**Supplementary file 1.** Source of the transcriptomes used for this analysis.

**Supplementary file 2.** Signal peptide predictions for human PNX precursors by different types of prediction software.

**Supplementary file 3.** Supermatrix used for the species tree in figure 3.

**Supplementary file 4.** Sequences of phoenixin precursors identified in different species with annotation.

**Supplementary file 5.** Data for the gene-structure of phoenixin precursors and accession number of the transcriptome and genome sequences used for the gene-structure analysis.

**Supplementary file 6.** Sequences of nesfatin-1 precursors identified in different species with annotation.

**Supplementary file 7.** Data for the gene-structure of NUCB precursors and accession number of the transcriptome and genome sequences used for the gene-structure analysis.

**Supplementary file 8.**  Sequences used for the phylogenetic analysis of SREB receptors in supplementary figure 5.

**Supplementary file 9.** Aligned and trimmed sequences used for the reconstruction of SREB receptors in supplementary figure 5.

**Supplementary file 10.** Nexus file of the tree shown in supplementary figure 5

**Supplementary file 11.** Raw data for the receptor deorphanisation in supplementary figure 6.

**Materials and Methods**

**Transcriptomic resources**

To identify the phyla to be included for the analysis of phoenixin and nesfatin-1 precursors, we performed an initial BlastP analysis in the NCBI database including metazoa, choanozoa, plants, fungi and prokaryotes. The phoenixin precursor (SMIM20) from human and *Nephrops norvegicus* (Yosten et al. 2013; Nguyen et al. 2018) and the nesfatin-1 precursor from human and *Drosophila melanogaster* were used as queries. No candidates were identified in plants and fungi. Thus, transcriptomes from different clades of metazoans, choanoflagellates, a filasterean and the flagellate *Tunicaraptor unikontum* were obtained from different public databases (see Supplementary file 1). We translated the transcripts into protein sequences with TransDecoder (TransDecoder; http://transdecoder.github.io/) with a minimum length of 50 amino acids. To assess the completeness of the transcriptomes, we ran BUSCO v5.2.1 [(Manni et al. 2021)](https://paperpile.com/c/LYvsPL/sS1HK) in protein mode and with the lineage set to ‘eukaryote’ with the database ‘eukaryota_odb10’ (Creation date of the database: Sep 2021, number of BUSCOs: 255).

**Phylogenomic analysis**

To build a tree representing the relationships of the 49 species studied, we carried out a phylogenomic analysis with the output of the BUSCO analysis. BUSCO datasets comprise genes evolving under “single-copy control” (Waterhouse et al. 2011) and are near-universally present as single-copy orthologs across lineages. The eukaryotic database has 255 single-copy orthologs. We aligned these orthologs from each species individually with MAFFT v7 using the iterative refinement method L-INS-i (Katoh et al. 2002). The alignment was trimmed with the TrimAl software using the gappy-out method [(Capella-Gutiérrez et al. 2009)](https://paperpile.com/c/LYvsPL/OOH0r). Then, we concatenated the trimmed alignments with FASconcatG [(Kück and Longo 2014)](https://paperpile.com/c/LYvsPL/m0eho), to assemble a concatenated supermatrix of 114,163 amino acid positions (Supplementary file 3). To build a species tree, we used IQ-TREE2 with the maximum-likelihood method under the LG+G4 model (Nguyen et al. 2015). The tree was rooted in the filasterean+*Tunicaraptor* clades. It is important to note that this phylogenomic analysis does not account for compositional bias and has been run with a homogeneous model (LG) only. The tree is merely used as a guide to map the evolutionary pattern of phoenixin, nesfatin-1 and the GPR173 across the species tree.

**Phoenixin and nesfatin-1 precursor identification and alignment**

We identified the phoenixin precursor sequences by using the phoenixin precursor (SMIM20) from human and *Nephrops norvegicus* [(Yosten, Lyu, et al. 2013; Nguyen et al. 2018)](https://paperpile.com/c/LYvsPL/U7vcG+mRWr) as queries. To search for nesfatin-1 precursor sequences, we used the human and *Drosophila melanogaster* precursors as queries [(Zandawala et al. 2017)](https://paperpile.com/c/LYvsPL/xJGpa). We used a BlastP search with an e-value of 1e-2 as the threshold to collect homologous sequences. In order to minimise the possibility of false positives, we manually curated the sequence list. After testing different signal peptide prediction (SignalP 3.0, SignalP 4.1, SignalP 5.0, SignalP 6.0) and subcellular localization tools (DeepLoc 1.0, TargetP 2.0) with the human PNX precursor we decided to use signalP-3.0 to detect signal peptides. The initially detected precursors were then used as new query sequences in a second BlastP search to detect potentially hidden orthologs. To align the full-length precursors and predicted mature peptides derived from them, we used MUSCLE [(Edgar 2004)](https://paperpile.com/c/LYvsPL/qfGAh). The lists of the sequences used for these alignments are available in Supplementary file 4 and Supplementary file 6.

**Gene-structure analyses of phoenixin and nesfatin-1 precursor sequences**

In all the species in which we identified phoenixin and/or nefastin-1 precursors, we also searched for the corresponding genes with Blast in the GenBank database. For gene structure analysis, we selected at least one species from each of the major clades of metazoans and the choanoflagellate *S. rosetta* and we retrieved the transcripts and genomic regions. We used the tool Splign (Kapustin et al., 2008) to determine the exon/intron structure of the genes (<https://www.ncbi.nlm.nih.gov/sutils/splign/splign.cgi>). Based on these data, the gene-structure diagrams were drawn in Adobe Illustrator CS6. The output of the Splign analysis is available in Supplementary files 5 and 7 (for phoenixin and nesfatin-1, respectively).

**GPR173 identification and phylogenetic analysis**

To identify GPR173 receptors, we obtained a database of vertebrate SREB sequences, including GPR173, GPR85 and GPR27 from [(Breton et al. 2021)](https://paperpile.com/c/LYvsPL/hHqjM). From these sequences, we produced a Hidden Markov Model (HMM) and used this to mine the 49 species investigated. HMM models were run in HMMR3 with an e-value of 1e-15. The same SREB sequences were used to carry out similarity-based searches using BlastP with an e-value cutoff of 1e-15. We merged these two databases and ran CD-Hit [(Fu et al. 2012)](https://paperpile.com/c/LYvsPL/D75h), to eliminate redundant sequences (at a 99% threshold). To identify the sequences that are closely related to the GPR173 sequences, we ran a cluster-based analysis in CLANS [(Frickey and Lupas 2004)](https://paperpile.com/c/LYvsPL/PqMX).The CLANS analysis is available as Supplementary figure 4. To identify clusters, we used the convex-clustering option with 100 jackknife replicates. The SREB receptors are extremely well conserved and form an easily recognisable cluster. To analyse the phylogeny of SREB receptors, the cluster containing these receptors together with monoaminergic receptors were parsed and used for tree building. We aligned the sequences with MAFFT version 7, with the iterative refinement method E-INS-i. Alignments were trimmed with TrimAl in gappy-out mode (Capella-Gutiérrez et al. 2009). To calculate maximum-likelihood trees, we used IQ-tree2 with the LG+G4 model. To calculate branch support, we ran 1,000 replicates with the aLRT-SH-like and aBayes methods [(Minh et al. 2020)](https://paperpile.com/c/LYvsPL/D2o0G). The sequences used for the phylogenetic analysis are available in Supplementary file 8, the trimmed alignment is available in Supplementary file 9. The raw nexus tree of the SREB receptors is available in Supplementary file 10.

**GPR173 deorphanisation assays**

We ordered the synthetic mature peptide PNX14 from GenScript with a purity of >95%. The receptors GPR173, GPR85 and GPR27 were purchased from the GenScript GenEZ human ORFs database (Accession No. NM_018969.6, NM_001146266.1 and NM_018971.2 respectively) and cloned into a pcDNA3.1(+) vector with EcoRV enzyme and a blunt cloning strategy. We expressed the receptors in HEK293 cells that were stably expressing the calcium-sensitive bioluminescent reporter GFP-aequorin fusion protein (G5A). This cell line was purchased from Angio-proteomie (CAT no. cAP-0200GFP-AEQ-Cyto). The HEK293-G5a cells were maintained at 37 °C in an 5 % CO2 atmosphere, in 96 well-plates containing 100 μl of DMEM high glucose glutamax medium (Thermo; Cat. No. 10566016) supplemented with 10% foetal bovine serum (Thermo; Cat. No. 10082147). Upon reaching confluency of approximately 85%, we transfected the cells with the plasmid containing the receptor to be tested and a plasmid containing the promiscuous Gαqi9 [Addgene; Cat. No. 125711 [(Masharina et al. 2012)](https://paperpile.com/c/LYvsPL/LvECi)] or Gαqs5 [Addgene; Cat. No. 24498 ] [(Conklin et al. 1996)](https://paperpile.com/c/LYvsPL/MVkpo).

Transfections were carried out with 60 ng of each plasmid and 0.35 μl of the transfection reagent Transfectamine 5000 (AAT-bioquest; Cat. No. 60022). Two days post-transfection, we removed the culture medium and substituted it for fresh DMEM-medium supplemented with 4 mM coelenterazine-H (Thermo Fisher Scientific; Cat. No. C6780). After an incubation period of 3 hr, we exposed the cells to synthetic PNX-14 peptide diluted in DMEM-medium in concentrations ranging from 10−4 M to 10−6 M. Luminescence levels were recorded over a 60-second period in a FlexStation 3 Multi-Mode Microplate Reader (Molecular Devices).

We integrated the luminescence data over a 60-second measurement period. A minimum of two independent transfections with triplicate measurements were made for each concentration, and the average of each was used to normalise the responses. We normalised the responses to the maximum response obtained by the addition of 100 μM ATP in each experiment (100% activation) and to the response obtained with the vehicle media (0% activation). As positive control for the Gαqi/9 protein, we used the *Clytia hemisphaerica* MIH receptor and one of its MIH-peptide ligands, RPRYamide [(Quiroga Artigas et al. 2020)](https://paperpile.com/c/LYvsPL/y8FHw). As positive control for the Gαqs5 protein, we used the human serotonin receptor 4 (5-HTR4) purchased from the GenScript GenEZ human ORFs database (Accession No. NM_000870.6) and tested it with serotonin hydrochloride purchased from Sigma-Aldrich (Cat No.H9523). For the positive controls, responses were normalised to the maximum response obtained by the addition of the activating compound (i.e serotonin or RPRYamide peptide), dose-response curves were fitted with a four-parameter curve based on the normalised data from the average of three independent transfections using Prism 8 (GraphPad, La Jolla, USA). The raw data obtained from the deorphanisation assays shown in Supplementary Figure 6 are available in Supplementary file 11.
