# Supplementary figures and images for: Premetazoan Origin of Neuropeptide Signaling
Source: Mol Biol Evol. 2022 Mar 12;39(4):msac051. doi: 10.1093/molbev/msac051 (PMC9004410; doi:10.1093/molbev/msac051)

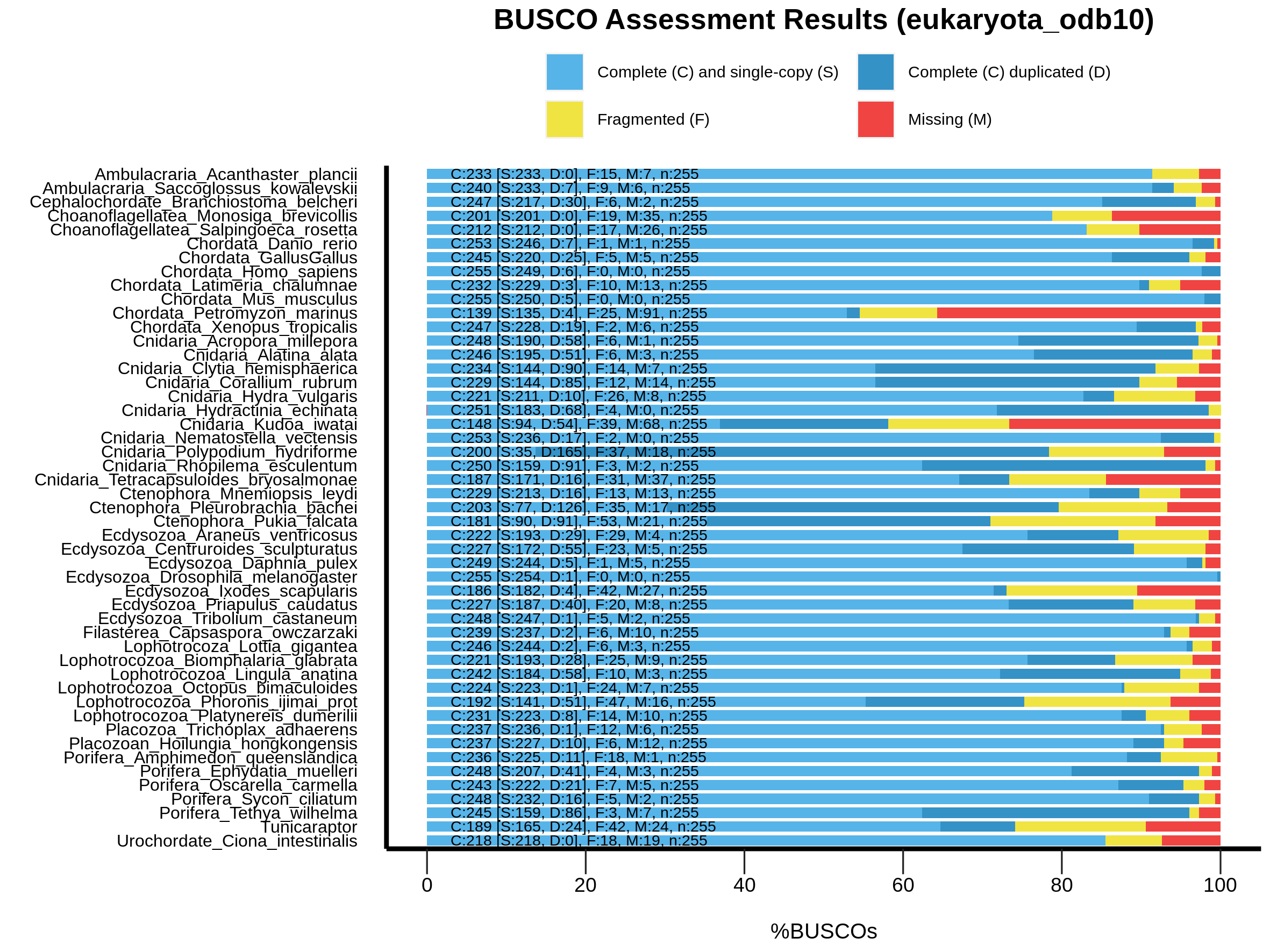

Supplement: msac051_Supplementary_Data [file msac051_supplementary_data.zip › Supplementary_Figure1_BUSCO.png]

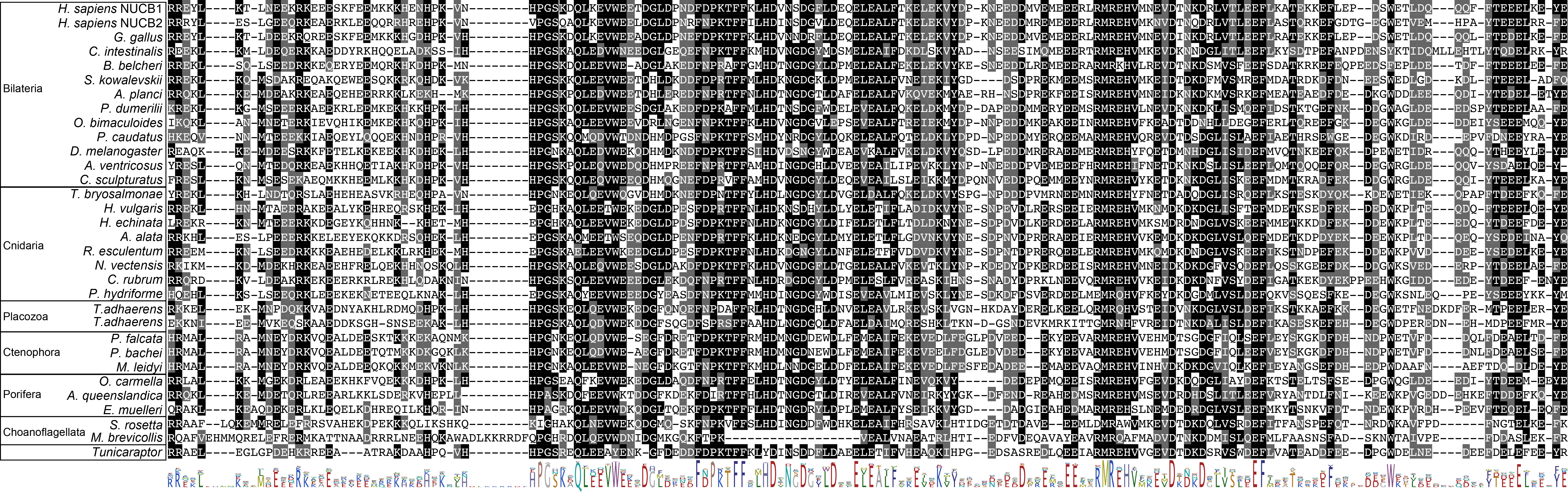

Supplement: msac051_Supplementary_Data [file msac051_supplementary_data.zip › Supplementary_Figure2.png]

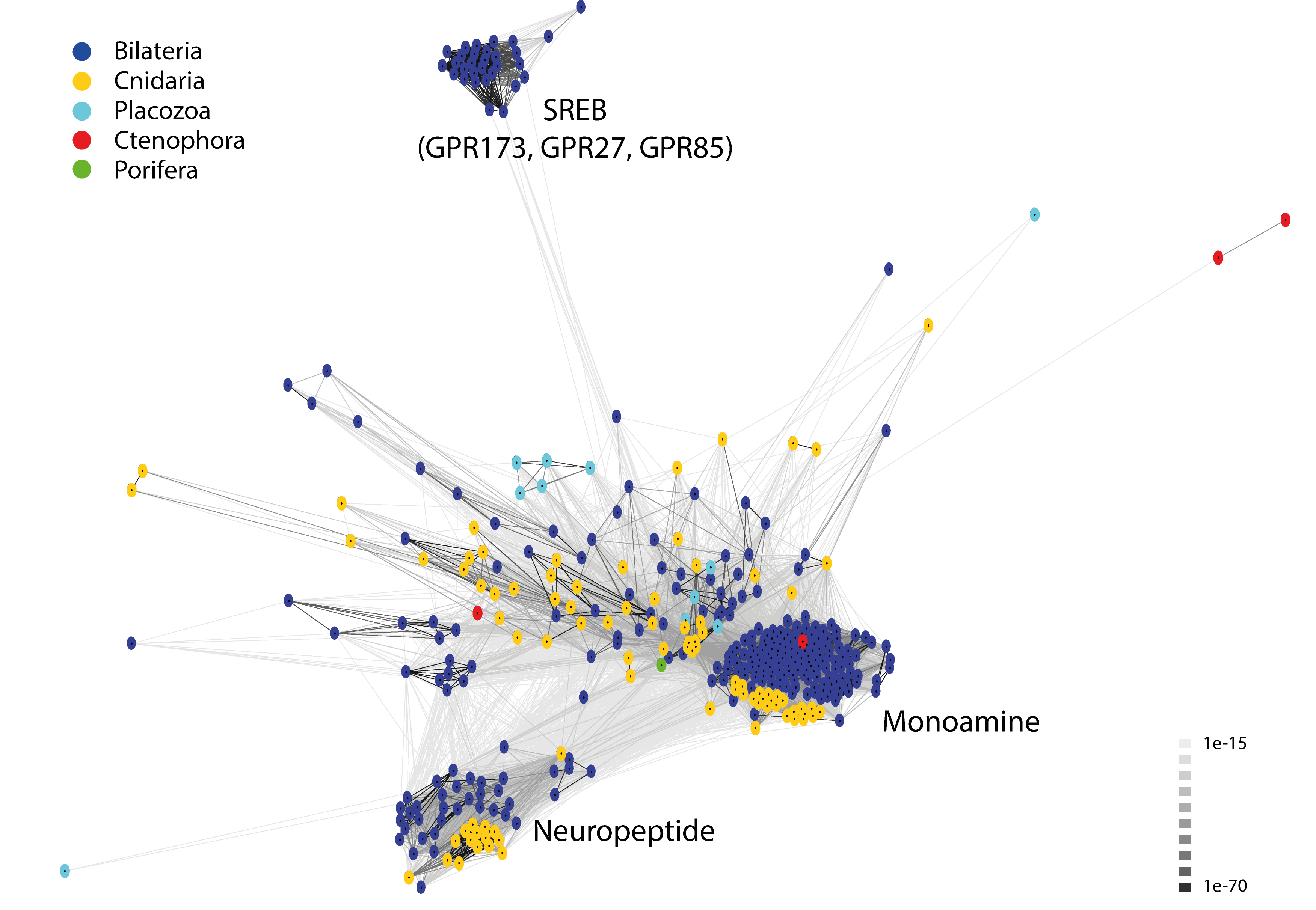

Supplement: msac051_Supplementary_Data [file msac051_supplementary_data.zip › Supplementary_Figure4_CLANSofSREBs.tif]

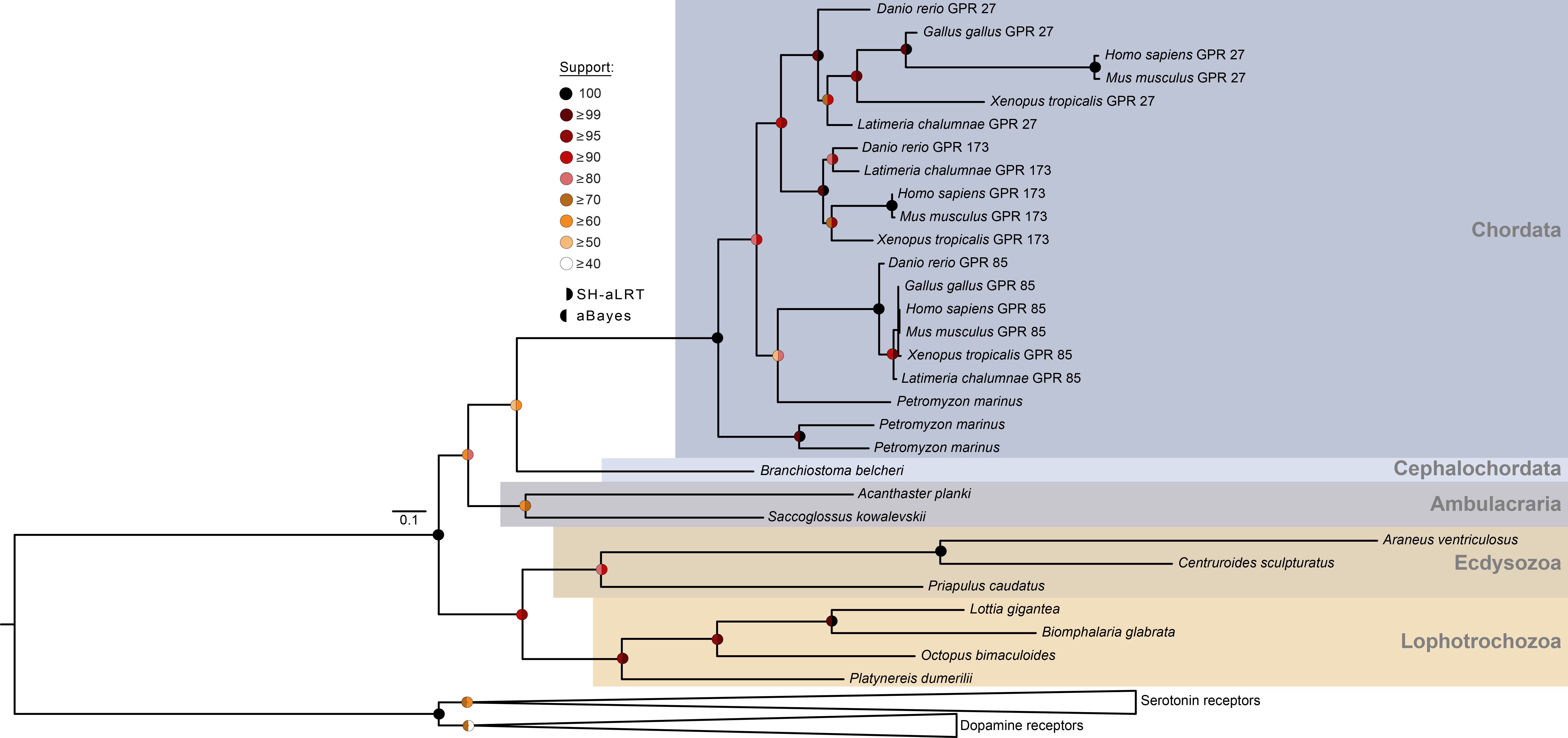

Supplement: msac051_Supplementary_Data [file msac051_supplementary_data.zip › Supplementary_Figure5_SREBtree.png]
