# Supplementary material for: Premetazoan Origin of Neuropeptide Signaling
Source: Mol Biol Evol. 2022 Mar 12;39(4):msac051. doi: 10.1093/molbev/msac051 (PMC9004410; doi:10.1093/molbev/msac051)

Color Align Conservation results

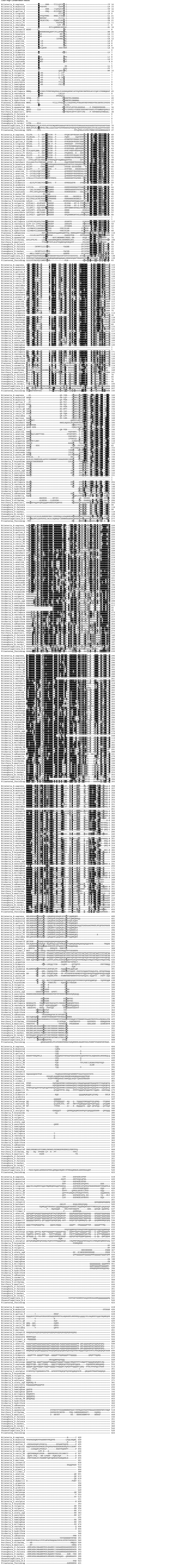

Supplement: msac051_Supplementary_Data [file Supplementary_Figure3_NUCB_full_alignment.pdf › Supplementary_Figure3_NUCB_full_alignment.pdf]
